# Complete mitochondrial genomes of two blattid cockroaches, *Periplaneta australasiae* and *Neostylopyga rhombifolia*, and phylogenetic relationships within the Blattaria

**DOI:** 10.1371/journal.pone.0177162

**Published:** 2017-05-09

**Authors:** Jinnan Ma, Chao Du, Chuang Zhou, Yongmei Sheng, Zhenxin Fan, Bisong Yue, Xiuyue Zhang

**Affiliations:** 1 Key Laboratory of Bio-resources and Eco-environment, Ministry of Education, College of Life Sciences, Sichuan University, Chengdu, China; 2 Sichuan Key Laboratory of Conservation Biology on Endangered Wildlife, College of Life Sciences, Sichuan University, Chengdu, China; 3 Sichuan Key Laboratory of Medicinal American Cockroach, Chengdu, China; Agriculture and Agri-Food Canada, CANADA

## Abstract

Complete mitochondrial genomes (mitogenomes) of two cockroach species, *Periplaneta australasiae* and *Neostylopyga rhombifolia*, 15,605 bp and 15,711 bp in length, respectively, were determined. As reported for other cockroach mitogenomes, the two mitogenomes possessed typical ancestral insect mitogenome gene composition and arrangement. Only several small intergenic spacers were found: one, which was common in all sequenced cockroach mitogenomes except for the genus *Cryptocercus*, was between tRNA-Ser (UCN) and ND1 and contained a 7bp highly conserved motif (WACTTAA). Three different types of short tandem repeats in the *N*. *rhombifolia* control region (CR) were observed. The homologous alignments of these tandem repeats with other six cockroach mitogenome CRs revealed a low similarity. Three conserved sequence blocks (CSB) were detected in both cockroach mitochondrial CRs. CSB1 was specific for blattinine mitogenomes and was highly conserved with 95% similarity, speculating that this block was a possible molecular synapomorphy for this subfamily. CSB3 located nearby downstream of CSB1 and has more variations within blattinine mitogenomes compared with CSB1. The CSB3 was capable of forming stable stem-loop structure with a small T-stretch in the loop portion. We assessed the influence of four datasets and two inference methods on topology within Orthopteroidea. All genes excluding the third codon positions of PCGs could generate more stable topology, and higher posterior probabilities than bootstrap values were presented at some branch nodes. The phylogenetic analysis with different datasets and analytical methods supported the monophyly of Dictyoptera and supported strongly the proposal that Isoptera should be classified as a family (Termitidae) of the Blattaria. Specifically, *Shelfordella lateralis* was inserted in the clade *Periplaneta*. Considering the K2P genetic distance, morphological characters, and the phylogenetic trees, we suggested that *S*. *lateralis* should be placed in the genus *Periplaneta*.

## Introduction

Cockroaches are a various insect of some 4,600 species and 460 genera are described now [1]. A relatively small number, considering the large number of species, of cockroaches are known as pests [2]. These pest cockroaches cause health problems, such as asthma and allergies, as well as economic costs for controlling them [3]. The cockroach (Insecta: Blattaria) *Periplaneta australasiae* and *Neostylopyga rhombifolia* belong to the subfamily Blattinae family Blattidae. They both are abundant and widely distributed urban pests [4]. Within the Blattinae, the species of the genus *Neostylopyga* are unique with the tegmina and wings strongly reduced or absent [5]. The Australian cockroach (*Periplaneta australasiae*) is a tropical cockroach, which is similar to type species, *Periplaneta americana*, in morphology, except for the pattern on the pronotum. There are approximately 324 species and 24 genera of Blattinae [6], as most of them lack of molecular data, the phylogenetic relationships among them are still contrasty [7].

Dictyoptera includes the Blattaria, Isoptera and Mantodea, and its monophyly was confirmed by molecular phylogeny [7] and morphology [8]. However, some studies showed Isoptera was nested within Blattaria (Blattaria and Isoptera together are called Blattodea) [9–10]. Although there are many studies on the relationships among the dictyopteran subgroups, the consensus about the relationships among different dictyopteran lineages has not been reached. Most studies supported the Blaberidae and Blattellidae clustered into a clade [11–13], but its position relationship within Blattaria is still disputed [11–12, 14]. Besides, a consensus about the relationships among remaining dictyopteran (Polyphagidae, Nocticolidae, Blattidae, Lamproblattidae, Tryonicidae) lineages has not yet been reached: In the molecular studies, Maekawa and Matsumoto [11] concluded Blattidae was a sister group to Blaberidae + Blattellidae and the Polyphagidae was at the base of Blattaria, while Legendre et al [15] supported the basal position of Blattodea was Blaberidae + Blattellidae, and that Polyphagidae + Nocticolidae was the sister group of all other remaining Blattodea, and Djernæs et al found that Lamproblattidae + Blattidae was the sister clade to (Nocticolidae + Polyphagidae) + (Cryptocercidae + Isoptera) [7]. Different discussions existed in morphological studies, based on numerical cladistic analyses used characters of the female genitalia, wings, and some other organs. Grandcolas [16] inferred the topology among cockroaches was Blattidae + (Polyphagidae + (Blaberidae + Blattellidae)). While the analysis focus on the asymmetrical male genitalia formed the topology of Blattidae + (((Polyphagidae + Lamproblattidae) + (Cryptocercidae + Isoptera)) + (Blaberidae + Blattellidae)) [12].

Insect mitogenome is typically 15–18 kb in size which encodes 13 protein-coding genes (PCGs) plus 22 transfer and 2 ribosomal RNA genes [17]. In addition, there are a variety of noncoding structural features of which the largest is known as the A+T-rich region, including some conserve structural elements [18]. Owing to maternal inheritance, a relatively rapid evolutionary rate, and lack of intermolecular recombinations, mitochondral DNA has been used widely in studies of population structures, molecular evolution, phylogeography, and phylogenetic relationships [9, 19–21]. Complete mitochondrial genomes are not only more informative than single or multi-genes, but also provide several genome-level characters, such as gene content and gene organization, genetic codon variation, tRNA and rRNA gene secondary structures, and pattern of controlling replication and transcription [22]. However, only 12 complete mitochondrial genomes are currently available for Blattaria. Considering the diversity of the Blattaria, which contains 4,600 described species, the existing full-length mitogenome sequence information for the Blattaria remains rather limited.

In this study, we sequenced and annotated the complete mitochondrial genomes of *P*. *australasiae* and *N*. *rhombifolia*, identified double control regions in both species, and compared various motifs to the other available blattarian mitogenomes. We reconstructed a phylogeny of Orthopteroidea to determine the relationships within Dictyoptera especially within Blattaria at the family level by using these two new mitogenomes in addition to the previously published mitogenomes of Orthopteroidea. We also assessed the influence of data types, phylogeny inference methods, exclusion and inclusion third codon positions of PCGs on topology and nodal support within Dictyoptera. In order to avoid the taxonomical confusions, we essentially followed the taxonomy system for the cockroaches, proposed by Louis [6] including six recognized families: Blattidae, Polyphagidae, Cryptocercidae, Blattellidae, Nocticolidae, and Blaberidae.

## Materials and methods

### Sample and DNA extraction

Cockroaches (Insecta: Blattaria) *Periplaneta australasiae* and *Neostylopyga rhombifolia* are all abundant and widely distributed urban pests [2]. These two cockroaches closely associated with human food, storage, harborage, and conditions provided by humans. They even cause health problem, such as asthma and allergies [2]. People always try to catch or kill these cockroaches for controlling their number in house. In this study, *Periplaneta australasiae* and *Neostylopyga rhombifolia* were collected respectively in Dongguan in Guangdong Province, and in Yulin in Guangxi Province on February 2016. Both specimens were collected in volunteers’ homes. We thanked both volunteers, Shilin He and Wujiao Li, in the Acknowledgments section. No specific permissions were required for these locations and this study did not involve endangered or protected species.

The fresh materials were preserved in 100% ethanol and stored in a -20°C refrigerator. Whole-genomic DNA was extracted from muscle tissue with the TIANamp Genomic DNA kit (TIANGEN, Beijing, China).

### PCR amplification and sequencing

The research follows Simon et al amplification and sequencing methods [23]. The primers were designed from aligned conserved nucleotide sequences of *Periplaneta americana* [10] and *Periplaneta fuliginosa* [24]. Then, based on the obtained sequences, species-specific primers were designed using Primer Premier 5.0 to amplify the overlapping fragments. Primer sequences and locations for each PCR are listed in Table 1. Primers Nr1F and Pa1F were from Du et al [25]. Primers Nr9F, Nr9R, Nr10F, and Pa10F were from Xiao et al [10]. Within each PCR product, the full double-stranded sequence was determined by primer walking (PTC-100 thermal cycler, BioRad, Hercules, CA). The PCR was performed using Vazyme Taq enzyme with the following cycling conditions: an initial denaturation for 5 min at 94°C, followed by 35 cycles of denaturation for 30s at 94°C, annealing for 30 s at 51–62°C (depending on primer combinations), elongation for 1–3 min (depending on putative length of the fragments) at 72°C, and a final extension step of 72°C for 10 min. The PCR products were assessed by electrophoresis in a 1.5% agarose gel and were stained by double-stranded DNA binding fluorescent dye (GoldView stain). The PCR products were purified using the DNA agarose gel extraction kit (OMEGA, China) and then sequenced from both directions on an ABI PRISM 3730 DNA sequencer by Tsingke Biotechnology Company (Chengdu, China).

**Table 1:** Primers used in the PCR amplification of *Periplaneta australasiae* (Pa) and *Neostylopyga rhombifolia* (Nr) mitogenomes.

Primer name	Upstream primers sequences (5’-3’)	Downstream primers sequences (5’-3’)	Anneal temperature (°C)	Extension time (Second)
**Nr1**	174-AAGCTAATGGGTTCATACC^a^	1616-TATGATGAAGGCATGAGCAGT	54	90
**Nr2**	1349-TACTCCTATAGAATTGCATTCTA	3778-CTTGCTTTCAGTCATCTGATG	53	150
**Nr3**	3332-TATTGCAGTTCCATCCTTACG	5362-GTAGTCCGTGGAATCCTGTTG	55	120
**Nr4**	4890-AATGATGACGAGATATTGTACG	6641-TGCTTGGTTTGGATACGA	54.5	110
**Nr5**	6118-TCCAGTTAAGGAAATGTGTAG	7374-CATCTACTTTGGTTACTGCA	51.3	80
**Nr6**	6872-AAACGAAATGAATAACAGACAGT	8481-AGATCTTGTAATATGGCGGC	55	100
**Nr7**	8087-TCACTGACACCACAAATCAGTA	9870-TTTTAATGTCAGAGGGTAGT	54.3	110
**Nr8**	9543-AAAGCACCTTCACAAACAGA	11030-AAAGTATGGGTGGAATGGAA	55	90
**Nr9**	10814-TGAGGACAAATATCATTTTGAGG^b^	13089-GGACGAGAAGACCCTATAGAGT^b^	59	140
**Nr10**	12757-CCGGTCTGAACTCAGATCATGT^b^	14643-TGCCAGCAGTTGCGGTTATAC	55	120
**Nr11**	14022-CGGTACAGCCACTATGTTACGACTT	406-TAAGAATAGCAATGTTGAGGAAGC	61.4	120
**Pa1**	174-AAGCTAATGGGTTCATACCa	1602-GGTTGACCAAGTTCAGCACGA	57	90
**Pa2**	1349-TACTCCTATAGAATTGCATTCTA	3778-CTTGCTTTCAGTCATCTGATG	53	150
**Pa3**	3536-GCTGCCGACGTTCTTCAT	5433-AGCTGCTGCTTCAAATCC	57	120
**Pa4**	5142-GCTATCCTTCTCGCTTCAGG	6646-TTTAGGTGGTTGATTTGGATA	54	90
**Pa5**	6159-AACTTATTACTTTAGCGGTTG	8487-ATGAGCGTTTAGGTAGACGAAGT	52.3	140
**Pa6**	7707-CTAATCCTAATCCATCTCAAC	9349-TTATGTTTTCAATATCGGGTT	54	100
**Pa7**	8820-TTGAACCTGAAACCGGAGCT	11725-TTGGGTTCGTGGTACAATAC	56	180
**Pa8**	11415-CATGAAGTGGAGCACGACCAG	13288-TTCTCGCATGTTTATCAAAAAC	54	120
**Pa9**	12757-CCGGTCTGAACTCAGATCATGT^b^	14643-TGCCAGCAGTTGCGGTTATAC	55	120
**Pa10**	14381-CCTCTAAAAAGACTAAAATACCGCC	532-GGAATCATCAGTGAAAGGGAGC	61.3	110

a. From Chao Du et al [25].

b. From Bo Xiao et al [10].

### Sequence analysis and annotation

DNA SeqMan program, which included in the Lasergene software package (DNAStar Inc., Madison, Wis.), was used to assemble sequences. The most transfer RNA inference was conducted using tRNAscan-SE [26] with a cove score cut off of 1. TrnS (AGN) of the two species and trnR of *N*. *rhombifolia* were routinely not found by tRNAScan-SE; they were identified by eye, through reference to secondary structure models for those genes from other blattarian insects. The secondary structures of tRNA genes were drawn using Adobe Illustrator CS6. The 13 protein-coding regions between tRNA were identified by ORF Finder implemented by NCBI website (http://www.ncbi.nlm.nih.gov/projects/gorf/) with invertebrate mitochondrial genetic codes. The rRNA gene boundaries were interpreted as the end of a bounding tRNA gene, and comparison of sequences with homologous regions of known blattarian mitogenomes was done using MEGA 5.0 [27]. The A+T content of nucleotide sequences, genetic distances, and relative synonymous codon usage (RSCU) were calculated using MEGA 5.0. The AT skewness was calculated according to the following formula: AT skew = [A-T] / [A+T] [28]. Secondary structures of repeat regions within the mitochondrial control region were inferred from the mfold web server [29] (http://mfold.rna.albany.edu/?q=mfold/DNA-Folding-Form). Tandem Repeat Finder v4.07 was used to confirm tandem repeats in A+T-rich regions [30].

### Phylogenetic inference

We used mtDNA sequences of 57 species taken from GenBank plus those of two additional species newly sequenced for this study. The list included 14 cockroaches, 17 termites, 8 mantids, 9 grasshoppers, 8 stick insects and a mantophasmatid. Mitochondrial genomes of two dragonflies (Odonata), *Brachythemis contaminata* and *Hydrobasileus croceus*, were used as outgroups (S1 Table). We set up four datasets with different gene content: nucleotide data of all genes (protein-coding, ribosomal RNA, and transfer RNA genes) (ALL-123), nucleotide data of all genes excluding the third codon sites of the protein-coding genes (ALL-12), nucleotide data of protein-coding genes (PCG-123), nucleotide data of protein-coding genes excluding third codon sites (PCG-12). Protein-coding genes, ribosomal RNA, and transfer RNA genes of these 57 species were extracted according to GenBank annotations using GenScalpel [31]. PCGs were aligned as DNA codons in MEGA 5.0, the unaligned and unmatched regions were selected and then back-translated into nucleotides and then deleted. The third codon positions of the 13 PCGs were excluded using DAMBE 6.4.42 [32]. Nucleotide sequences of RNA genes from the mitogenomes of the 59 species were aligned with MEGA 5.0, the unaligned and unmatched regions were removed, and then the concatenated nucleotide sequences were combined to the end of the aligned nucleotide of 13 PCGs respectively.

In order to reconstruct the phylogenetic relationships within Orthopteroidea, maximum likelihood (ML) and Bayesian inference (BI) were used to determine the effect of analytical method on topology and nodal support. The program Modeltest ver. 3.7 [33] was used with Akaike Information Criterion (AIC) [34] to calculate the substitution model selection. The GTR+I+G model was chosen as the best-fitting model for BI analysis. Then Bayesian inference (BI) analyses of nucleotides were performed with MrBayes 3.2.2 [35]. Four chains (one cold chain and three hot chains) ran in parallel for 10,000,000 generations, sampling every 100 generations and burn-in of 2500 trees. For maximum likelihood (ML) of nucleotide datasets, we implemented the GTR matrix in the PHYML online web server (http://www.atgc-montpellier.fr/phyml/) [36] with 1000 bootstrap replications. The phylogenetic trees were visualized by FigTree v1.4.0 [37] program with adjustable settings.

### Neighbor-joining analysis

To explore the phylogeny between *Periplaneta* species and *Shelfordella lateralis*, all of the haplotypes of the *Periplaneta* species and *Shelfordella lateralis* COI barcodes were taken from NCBI (Table 2). Sequences were trimmed to a final length of 598 bp. Pairwise nucleotide sequence divergences were estimated between all of the haplotypes of *Periplaneta* (five species) and *Shelfordella lateralis* COI barcodes sequences using the Kimura 2-parameter (K2P) model [38] implemented in MEGA 5.0 [27].

**Table 2:** K2P genetic distances between all of the haplotypes of *Periplaneta* (five species) and *Shelfordella lateralis* COI barcodes sequences.

	1	2	3	4	5	6	7	8	9	10	11	12	13	14	15	16	17	18	19	20	21
*Periplaneta americana* |JX402724.1|		0.009	0.010	0.004	0.009	0.009	0.009	0.009	0.003	0.010	0.010	0.009	0.010	0.010	0.015	0.015	0.015	0.015	0.018	0.018	0.015
*Periplaneta americana* |KF640070.1|	0.045		0.007	0.009	0.002	0.002	0.005	0.004	0.009	0.007	0.006	0.002	0.007	0.007	0.015	0.015	0.015	0.014	0.018	0.018	0.015
*Periplaneta americana* |KM577024.1|	0.052	0.027		0.009	0.007	0.007	0.007	0.007	0.009	0.003	0.002	0.007	0.003	0.003	0.015	0.015	0.015	0.015	0.017	0.017	0.015
*Periplaneta americana* |KM577049.1|	0.008	0.042	0.049		0.008	0.008	0.009	0.009	0.002	0.009	0.009	0.008	0.009	0.009	0.015	0.015	0.015	0.015	0.017	0.017	0.015
*Periplaneta americana* |KM577080.1|	0.043	0.003	0.029	0.040		0.003	0.005	0.004	0.008	0.007	0.007	0.003	0.007	0.007	0.015	0.015	0.015	0.015	0.018	0.018	0.015
*Periplaneta americana* |KM577135.1|	0.045	0.003	0.027	0.042	0.007		0.004	0.003	0.008	0.007	0.006	0.002	0.006	0.007	0.015	0.015	0.015	0.015	0.018	0.018	0.015
*Periplaneta americana* |KM577145.1|	0.045	0.014	0.031	0.042	0.015	0.014		0.004	0.009	0.007	0.007	0.004	0.007	0.007	0.015	0.015	0.015	0.014	0.017	0.017	0.015
*Periplaneta americana* |KM577147.1|	0.047	0.008	0.029	0.043	0.010	0.008	0.012		0.009	0.007	0.006	0.003	0.007	0.007	0.015	0.015	0.015	0.014	0.017	0.017	0.015
*Periplaneta americana* |KM577150.1|	0.007	0.042	0.049	0.002	0.040	0.042	0.042	0.043		0.009	0.009	0.008	0.009	0.009	0.015	0.015	0.015	0.015	0.017	0.017	0.015
*Periplaneta americana* |KM577152.1|	0.056	0.027	0.007	0.052	0.029	0.027	0.031	0.029	0.052		0.002	0.007	0.003	0.002	0.015	0.015	0.015	0.015	0.018	0.017	0.016
*Periplaneta americana* |KM577154.1|	0.052	0.024	0.003	0.049	0.026	0.024	0.027	0.026	0.049	0.003		0.006	0.002	0.002	0.015	0.015	0.015	0.014	0.017	0.017	0.015
*Periplaneta americana* |KM577156.1|	0.043	0.002	0.026	0.040	0.005	0.002	0.012	0.007	0.040	0.026	0.022		0.006	0.006	0.015	0.015	0.015	0.014	0.018	0.018	0.015
*Periplaneta americana* |KM577157.1|	0.054	0.026	0.005	0.051	0.027	0.026	0.029	0.027	0.051	0.005	0.002	0.024		0.002	0.015	0.015	0.015	0.014	0.017	0.017	0.016
*Periplaneta americana* lKM577126.1|	0.054	0.026	0.005	0.051	0.027	0.026	0.029	0.027	0.051	0.002	0.002	0.024	0.003		0.015	0.015	0.015	0.014	0.017	0.017	0.016
*Periplaneta australasiae* |AM114928.1|	0.124	0.122	0.120	0.122	0.124	0.122	0.118	0.118	0.122	0.126	0.122	0.120	0.122	0.124		0.002	0.013	0.011	0.016	0.016	0.016
*Periplaneta australasiae* |KX640825|	0.122	0.120	0.118	0.120	0.122	0.120	0.116	0.116	0.120	0.124	0.120	0.118	0.120	0.122	0.002		0.013	0.011	0.016	0.016	0.016
*Periplaneta brunnea* |AM114930.1|	0.120	0.118	0.120	0.116	0.120	0.120	0.112	0.114	0.116	0.122	0.118	0.118	0.118	0.120	0.101	0.099		0.013	0.016	0.016	0.017
*Periplaneta fuliginosa* |AB126004.1|	0.118	0.110	0.114	0.116	0.112	0.112	0.104	0.108	0.116	0.116	0.112	0.110	0.112	0.114	0.078	0.076	0.093		0.013	0.013	0.015
*Periplaneta japonica* |AM114929.1|	0.170	0.168	0.168	0.164	0.170	0.170	0.158	0.162	0.164	0.170	0.166	0.168	0.164	0.168	0.130	0.132	0.139	0.114		0.002	0.017
*Periplaneta japonica* |JQ350708.1|	0.168	0.166	0.166	0.162	0.168	0.168	0.156	0.160	0.162	0.168	0.164	0.166	0.162	0.166	0.128	0.130	0.137	0.112	0.002		0.017
*Shelfordella lateralis* |NC_030003.1|	0.126	0.127	0.133	0.126	0.131	0.129	0.129	0.126	0.126	0.141	0.137	0.127	0.139	0.139	0.130	0.128	0.163	0.130	0.158	0.156	

Pairwise nucleotide sequence divergence(s) are shown below and standard error estimate(s) are shown above the diagonal.

## Results

### Genome content and organization

**Fig 1:**
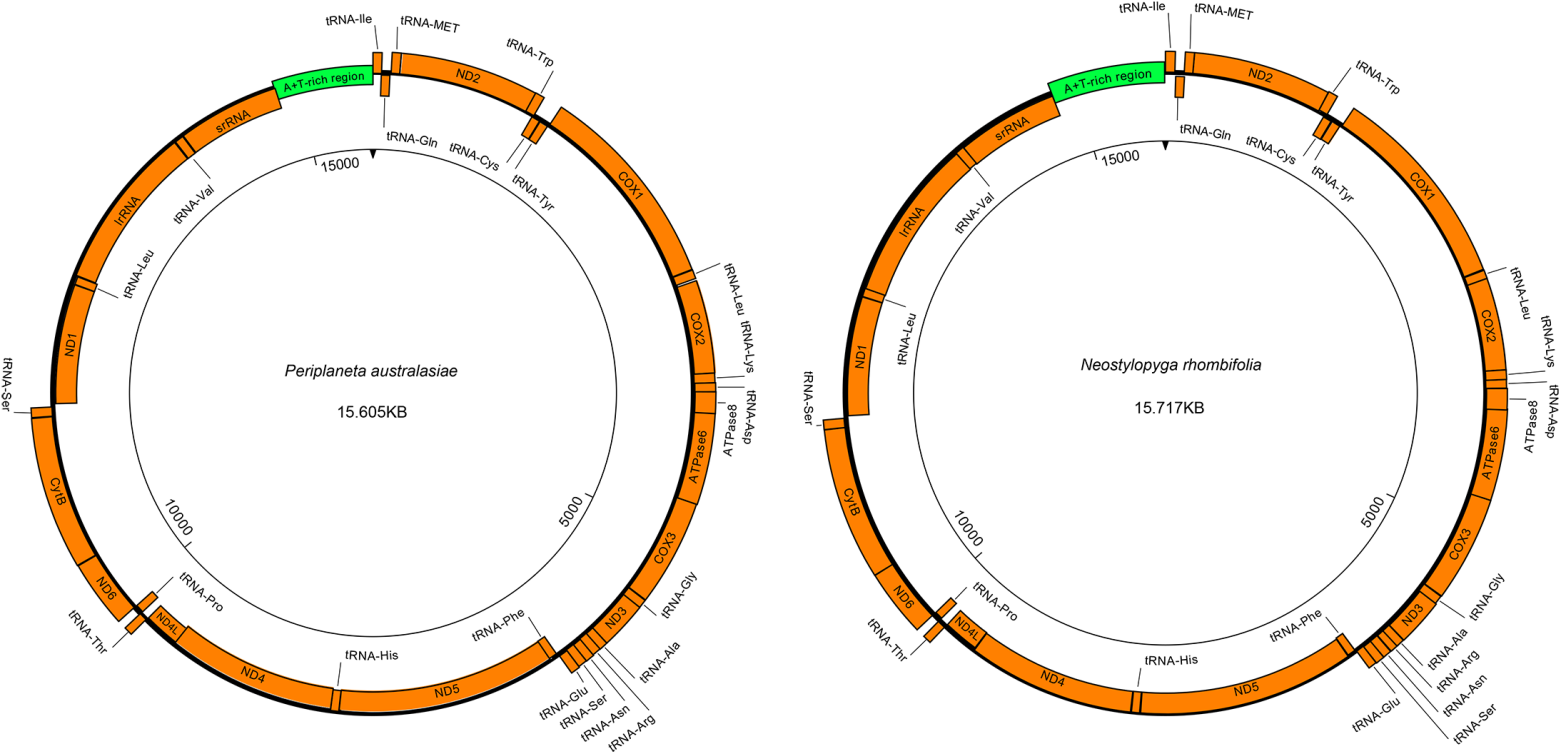
Mitochondrial gene maps of *Periplaneta australasiae* and *Neostylopyga rhombifolia*. Genes coded in the J-strand are inside of the circle. Gene coded in the N-strand are outside of the circle. COX1, COX2 and COX3 refer to the cytochrome C oxidase subunits; CytB refers to cytochrome B; ATPase6 and ATPase8 refer to ATP synthase subunits 6 and 8 genes; and ND1-ND6 and ND4L refer to the NADH dehydrogenase subunit 1–6 and 4L genes.

The mitochondrial genomes of *Periplaneta australasiae* and *Neostylopyga rhombifolia* were typical circular molecules, 15,605bp and 15,711bp in length, respectively, and were submitted to GenBank under the accession numbers KX640825 and KX640826. The sizes of the *P*. *australasiae* and *N*. *rhombifolia* mitogenomes were within the range of other blattarian mitogenomes, with the lengths ranging from 14,996 bp (NC_006076.1) to 17,340 bp (NC_029224.1) (S1 Table). The gene order and orientation of *P*. *australasiae* and *N*. *rhombifolia* mitogenomes were identical to those of other reported blattarian cockroach species (Fig 1 and Table 3) and had the ancestral insect gene composition and arrangement [23]. Additionally, as other dictyopteran insect mitogenomes, the nucleotide composition of the *P*. *australasiae* and *N*. *rhombifolia* mitogenomes had a high A+T bias of 74.9% and showed a skew of A’s (S1 Table).

**Table 3:** Annotation of *Periplaneta australasiae* (Pa) and *Neostylopyga rhombifolia* (Nr).

Gene	Strand	Location		Anticodon	Start codon	Stop Codon
		Pa	Nr		Pa/Nr	Pa/Nr
窗体顶端 tRNA-Ile	J	1–67	1–67	GAT		
tRNA-Gln	N	65–133	65–133	TTG		
tRNA-MET	J	141–207	141–207	CAT		
ND2	J	208–1236	208–1233		ATG/ATG	TAA/TAA
tRNA-Trp	J	1236–1306	1233–1303	TCA		
tRNA-Cys	N	1299–1366	1296–1365	GCA		
tRNA-Tyr	N	1376–1444	1384–1453	GTA		
COX1	J	1449–2984	1458–2996		TTG/TTG	TAA/TAA
tRNA-Leu(UUR)	J	2990–3055	3004–3067	TAA		
COX2	J	3072–3756	3069–3753		ATG/ATG	T-/T-
tRNA-Lys	J	3757–3827	3754–3824	CTT		
tRNA-Asp	J	3828–3892	3825–3888	GTC		
ATPase8	J	3893–4051	3889–4047		ATT/ATT	TAA/TAA
ATPase6	J	4045–4724	4041–4721		ATG/ATG	TA-/TAA
COX3	J	4725–5513	4721–5509		ATG/ATG	TAA/TAA
tRNA-Gly	J	5518–5582	5514–5578	TCC		
ND3	J	5583–5934	5579–5932		ATT/ATT	T-/TAG
tRNA-Ala	J	5935–6000	5931–5994	TGC		
tRNA-Arg	J	6000–6066	5994–6060	TCG		
tRNA-Asn	J	6066–6131	6065–6130	GTT		
tRNA-Ser(AGN)	J	6132–6198	6131–6198	GCT		
tRNA-Glu	J	6200–6269	6200–6263	TTC		
tRNA-Phe	N	6271–6337	6264–6329	GAA		
ND5	N	6338–8068	6331–8058		ATG/ATA	TAA/TAA
tRNA-His	N	8069–8134	8062–8127	GTG		
ND4	N	8137–9474	8134–9471		ATG/ATG	TAA/TAA
ND4L	N	9468–9752	9465–9749		ATG/ATG	TAA/TAA
tRNA-Thr	J	9755–9818	9753–9816	TGT		
tRNA-Pro	N	9819–9885	9817–9881	TGG		
ND6	J	9888–10387	9884–10386		ATT/ATT	TA-/TA-
CytB	J	10388–11519	10388–11519		ATG/ATG	T-/T-
tRNA-Ser(UCN)	J	11525–11595	11521–11591	TGA		
ND1	N	11621–12568	11609–12556		ATG/ATG	TAA/TAA
tRNA-Leu(CUN)	N	12572–12635	12560–12625	TAG		
lrRNA	N	12636–13943	12626–13921			
tRNA-Val	N	13944–14014	13922–13993	TAC		
srRNA	N	14015–14826	13994–14808			
A+T-rich region		14827–15605	14809–15711			

‘TA-’ and ‘T-’ refer to incomplete stop codons.

The relative synonymous codon usage (RSCU) value of *P*. *australasiae* and *N*. *rhombifolia* mitogenomes was summarized in S2 Table. All initiation and termination codons were included: the UUA (L) codon was used most frequently, followed by CGA (R) and ACA (T) in *P*. *australasiae*, and CGA(R) and GUA (V) in *N*. *rhombifolia*. The codon usage preference of A+T-rich over synonymous codon families, which played a major role in the A+T bias of the entire mitogenome. All codons ending with A or T outnumber those ending with C or G, except for the Ser family in *N*. *rhombifolia*, where the AGG was used more than the AGA codon (RSCU = 1.09 and 1.19, respectively).

### Protein-coding genes

A summary of the mitogenomes of *P*. *australasiae* and *N*. *rhombifolia* was given in Table 3. As ancestral insect mitogenomes, four PCGs (ND5, ND4, ND4L, and ND1) were coded on the minority strand (N-strand) and the remaining nine of the 13 PCGs were coded on the majority strand (J-strand). For *P*. *australasiae*, nine of 13 PCGs had ATG as the start codon, while COX1 utilized TTG, ATP8, ND6 and ND3 translated from ATT. For *N*. *rhombifolia*, ATG also was the most common start codon and occurred in the other eight PCGs except for COX1 (TTG), ATP8, ND6 and ND3 (ATT), and ND5 (ATA). COX1 uses TTG as the start codon which is an accepted conventional start codon for blattarian mitogenomes [10, 13, 39]. As for the termination codons, TAA and TAG were commonly used except COX2, ATP6, ND3, ND6, and CytB in *P*. *australasiae*, and COX2, ND6, and CytB in *N*. *rhombifolia*. For *P*. *australasiae*, COX2, ND3, and CytB stopped with T-, and ATP6 and ATP8 ending with TA-. For *N*. *rhombifolia*, COX2 and CytB stopped with incomplete T-, and ND6 used TA- nucleotides as incomplete stop codon.

### tRNA genes

The secondary structures of each tRNA are shown in S1 and S2 Figs. The typical set of 22 tRNA genes ranged from 64 to 71 bp in *P*. *australasiae* and from 64 to 72 bp in *N*. *rhombifolia*, which were conserved among insects [40]. Among these 22 tRNA genes of *P*. *australasiae* mitogenome, all can be detected by tRNAScan-SE with the exception of tRNA-Ser (AGN) due to the absence of DHU arm. In *N*. *rhombifolia*, besides tRNA-Ser (AGN), tRNA-Arg also can’t be spotted by tRNAScan-SE, in which the T-arm contained six paired nucleotides. These transfer RNAs were determined through comparison with previously published blattarian mitogenomes [10, 13, 41]. The secondary structures of most tRNA genes of the two mitogenomes were conversed except for tRNA-Lys, which only had four paired nucleotides in the anticodon arm. Findings showed twenty-nine mismatches of base pairs in *P*. *australasiae* tRNA genes, with twenty-four noncanonical matches of G-U base pairs. The other three U-U, one A-C, and one U-C base-pairings showed as mismatches in the stems of five different tRNAs (tRNA-Met, tRNA-Leu (CUN), tRNA-Ser (AGN), tRNA-Val, tRNA-Trp). In *N*. *rhombifolia*, there were five more unmatched base pairs in the tRNA genes than in *P*. *australasiae*. Twenty-eight of the mismatches in *N*. *rhombifolia* were G-U pairs; the remaining six mismatches were as follows: two U-U mismatches in tRNA-Leu (CUN), one U-U and one A-A mismatch in tRNA-Ser (AGN), one A-C mismatch in tRNA-Met and one U-C mismatch in tRNA-Trp.

### Non-coding regions

**Fig 2:**
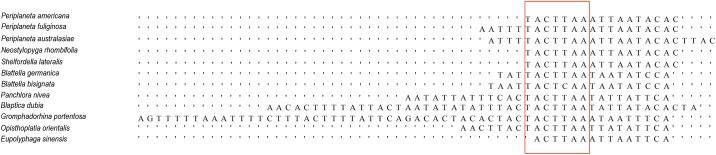
Alignment of intergenic spacer sequences (WACTTAA) in twelve cockroach mitogenomes. Consensus bases are shown in red color. The alignments are generated by plotting the identities to a standard as a dot.

Generally, the insect mitogenomes display the evolutionary economic perspective, but large intergenic spacers are existing in some insects [42]. Nevertheless, the complete genomes of *P*. *australasiae* and *N*. *rhombifolia* only contained a few short non-coding fragments and no long intergenetic spacers were found. The two longest intergenic spacers regions of more than 10 bp for *P*. *australasiae* were between tRNA-Leu (UUR) and COX2 (16bp) and between tRNA-Ser (UCN) and ND1 (25bp). *N*. *rhombifolia* had two relatively large intergenic spacers: one 18bp long was located between tRNA-Cys and tRNA-Tyr and another 17bp was at the same position as for the *P*. *australasiae* mitogenome (between tRNA-Ser (UCN) and ND1). We aligned all blattarian mitogenomes reported and found that, except for the genus *Cryptocercus*, the intergenic spacer between tRNA-Ser (UCN) and ND1 appeared in all sequenced blattarian mitogenomes and ranged from 15 bp in *Eupolyphaga sinensis* (NC_014274.1) to 58 bp in *Gromphadorhina portentosa* (NC_030001.1). These intergenic spacer sequences showed a 7bp highly conserved motif (WACTTAA) (Fig 2), which can be deemed to be the binding site of the transcription termination [43].

**Fig 3:**
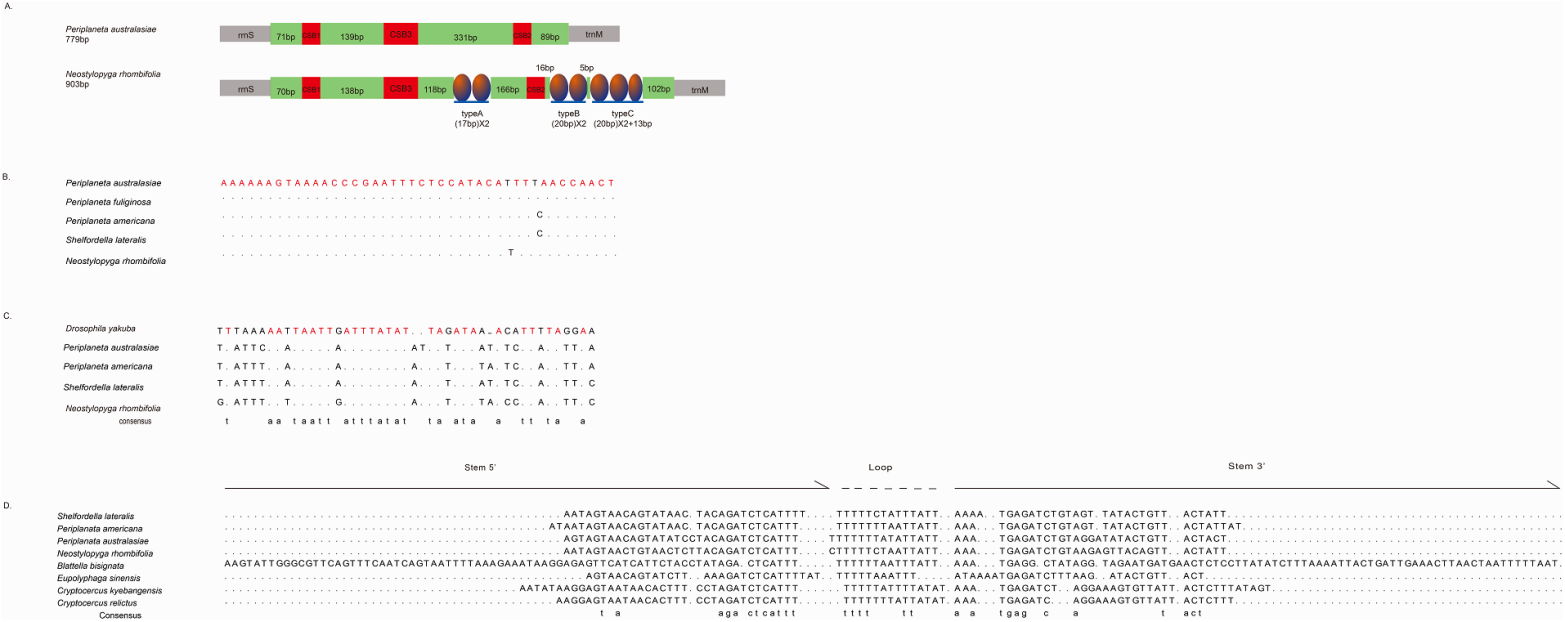
**(A) Organization of the A+T-rich region of *Periplaneta australasiae* and *Neostylopyga rhombifolia*. (B) Alignment of Conserved Sequence block1 in the A+T-rich regions of Blattinae. (C) Alignment of Conserved Sequence block2 in the A+T-rich regions of in Blattinae. (D) Alignment of Conserved Sequence block3 (stem-loop structures) in the A+T-rich regions of Blattaria.** An oval indicates a tandem repeat sequence. A colored box shows the non-repeat region, and a red box shows the conserved sequences. Consensus bases are shown in red color. The alignments are generated by plotting the identities to a standard as a dot and a gap as a dash.

The control region (CR) was thought to have played an important role in regulating the mtDNA transcription and replication [44–45]. The lengths of the CR of *P*. *australasiae* and *N*. *rhombifolia* were 779bp and 903bp, with AT contents of 81.6% and 80.0%, respectively. Three different types of short tandem repeats in *N*. *rhombifolia* CR were observed. The repeats were located closely to tRNA-Met which consisted of two full A type units, two full B type units, two full C type units, and partial C unit (Fig 3A). In insect mitochondrial control regions, low levels of primary sequence similarity across taxa have led to the suggestion of conserved structural elements [18]. Zhang & Hewitt [18] summarized that the structural elements among control regions were as follows: a long sequence of thymines, a [TA(A)]_n_ stretch between the poly T stretch, a highly conserved secondary structure, and conservative structure flanking the stem-loop structures. Three conserved sequence blocks were also identified in the *P*. *australasiae* and *N*. *rhombifolia* CRs: blocks 1, 2 and 3 (Fig 3). It is worth noting that these conserved blocks are spread through the whole A+T-rich region.

**Fig 4:**
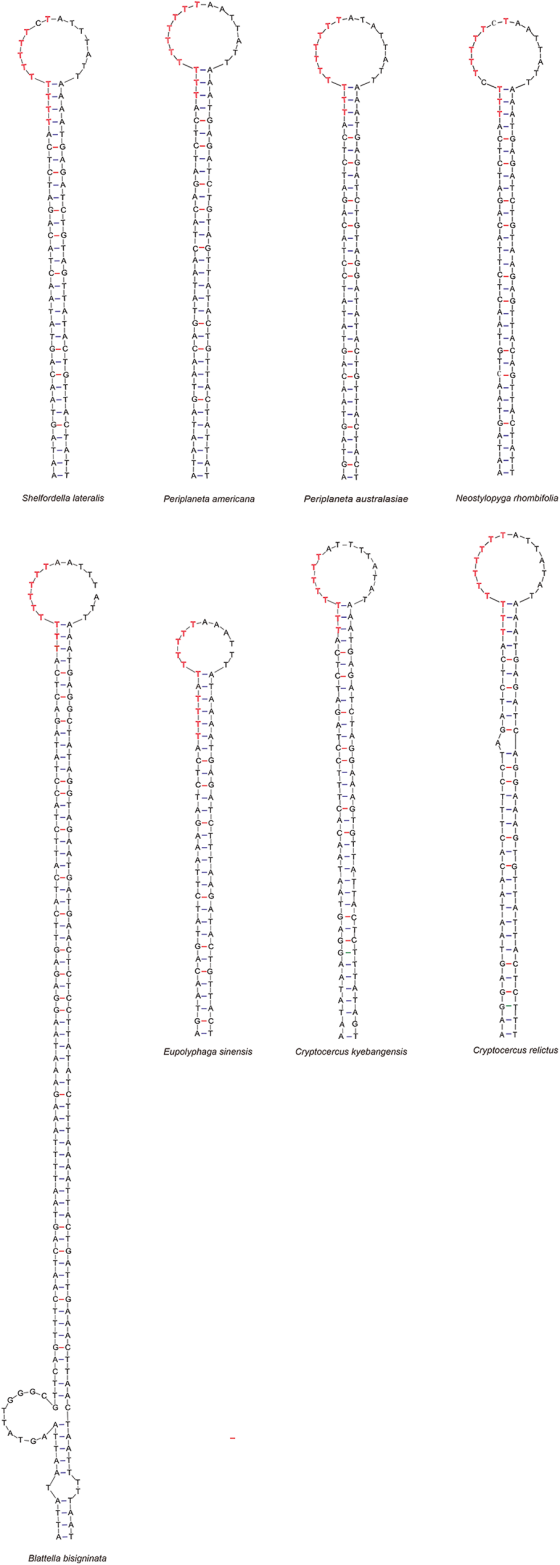
Possible conserved secondary structures of CSB3 in the mitochondrial control regions of Blattaria. The nucleotides highlighted in red represent the location of the T-stretch.

Conserved sequence block 1 (CSB1) was located closely downstream of the tRNA-Ile gene. This block was highly conserved within the Blattinae with only one nucleotide variation, or 95% similarity (Fig 3B), and it has not been found in other insect mitogenomes. Another conserved sequence block (CSB2) was identified by aligning with the [TA(A)]_n_ sequence described by Zhang et al [46]. The motif has the similar core sequence 5’-A…TAATTTA…TT…ATA…ACATTT-3’ which resembles the template stop signals for D-loop syntheses in human and mouse mtDNA [47]. The CSB3 was located nearby the downstream of CSB1 and has more variations (Fig 3D) compared with CSB1, which was a major stem and loop (or hairpin) found in the A+T-rich region (Fig 4). The stem ranged in size from 30 paired bases in *Eupolyphaga sinensis* to 77 paired bases in *Blattella bisignata* and the loop from 11 bp in size in *Eupolyphaga sinensis* to 15 bp in *P*. *australasiae* and *N*. *rhombifolia*. In addition, small T-stretches were observed in the loop portion of hairpin structures (Fig 4).

### Phylogenetic analyses

**Fig 5:**
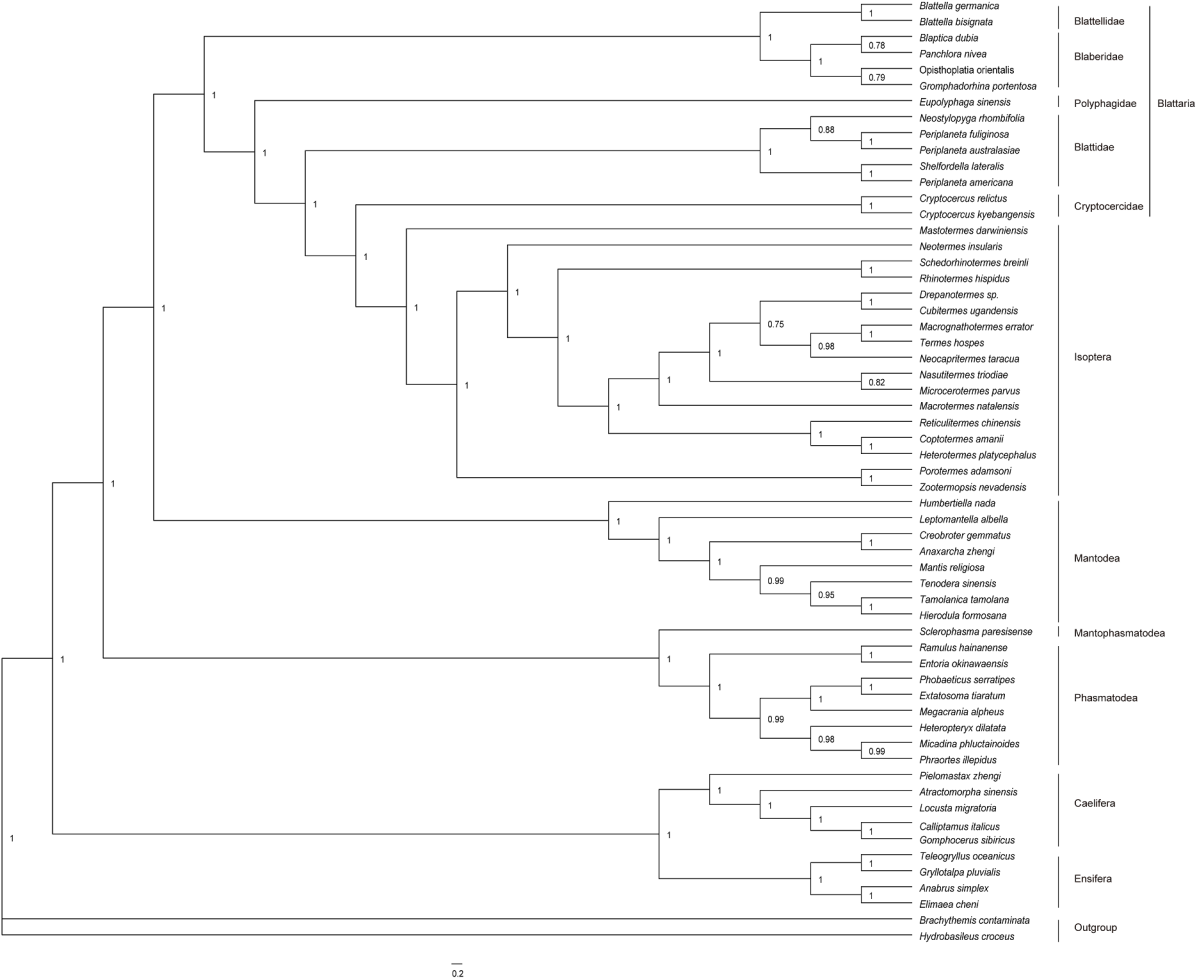
Bayesian phylogenetic tree based on the nucleotide data of all genes excluding the third codon sites of the protein-coding genes (BI-ALL-12). Numbers abutting branches refer to Bayesian posterior probabilities (BPP). Two odonatan insects *Brachythemis contaminata* and *Hydrobasileus croceus* were used to root the tree as outgroups.

**Fig 6:**
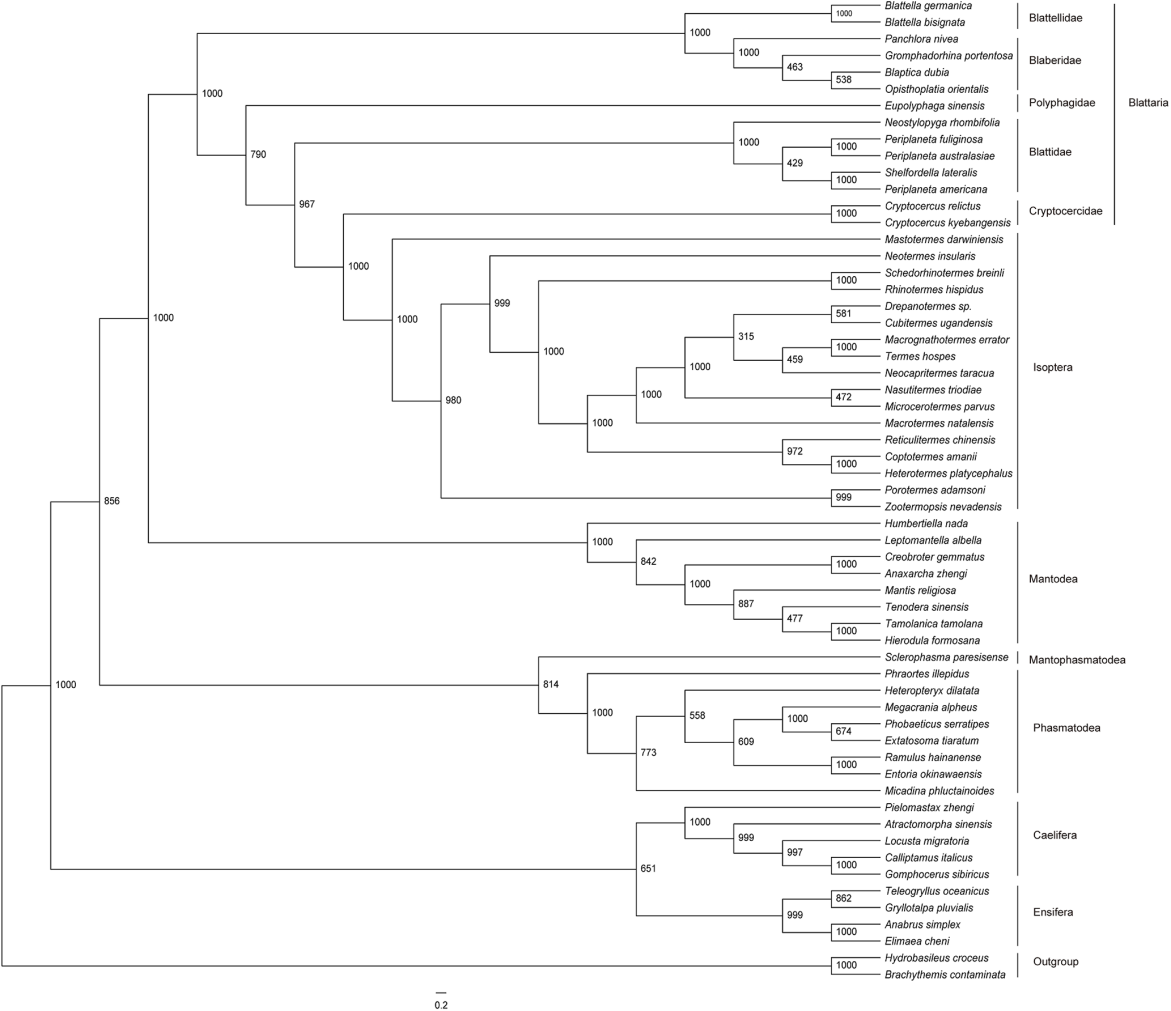
Maximum likelihood phylogenetic tree based on the nucleotide data of all genes excluding the third codon sites of the protein-coding genes (ML-ALL-12). Numbers abutting branches refer to bootstrap proportions (BSP). Two odonatan insects *Brachythemis contaminata* and *Hydrobasileus croceus* were used to root the tree as outgroups.

The phylogenetic relationships were analyzed based on four datasets using both ML and BI methods. The results of analysis of the ALL-12 dataset are presented in Figs 5 and 6, and the results of other analyses are presented in S3–S8 Figs. The topology and nodal support are sensitive to different datasets and inference methods. The major effect of the optimality criteria was seen at the interordinal level. In ML analyses (except ALL-12) (S4, S6 and S8 Figs), Orthoptera was paraphyletic, with Ensifera being sister to Phasmatodea. Besides, for the PCG-12 dataset, Mantophasmatodea had a sister relationship with Dictyoptera in ML analysis, whereas Mantophasmatodea clusters with Phasmatodea when Bayesian inference were used in analysis (S7 and S8 Figs). With regard to the nodal support, the posterior probabilities in BI analyses were generally higher than bootstrap values in ML analyses in some branch nodes. Different mitogenome data did not appear to affect support values much, but they did slightly affect topology at the interfamily level. When 13 protein-coding genes were analyzed as a single partition (PCG-123), Blattellidae + Bleberidae was clustered with Blattidae, but which was basal clade of Blattodea in ALL-123 analyses (S3–S6 Figs). The position of *Eupolyphaga sinensis* is variable, either as first branch within Blattodea (ML-PCG-12) (S8 Fig) or as sister to Blattidae + (Cryptocercidae + Isoptera) (ALL-12) (Figs 5 and 6).

**Fig 7:**
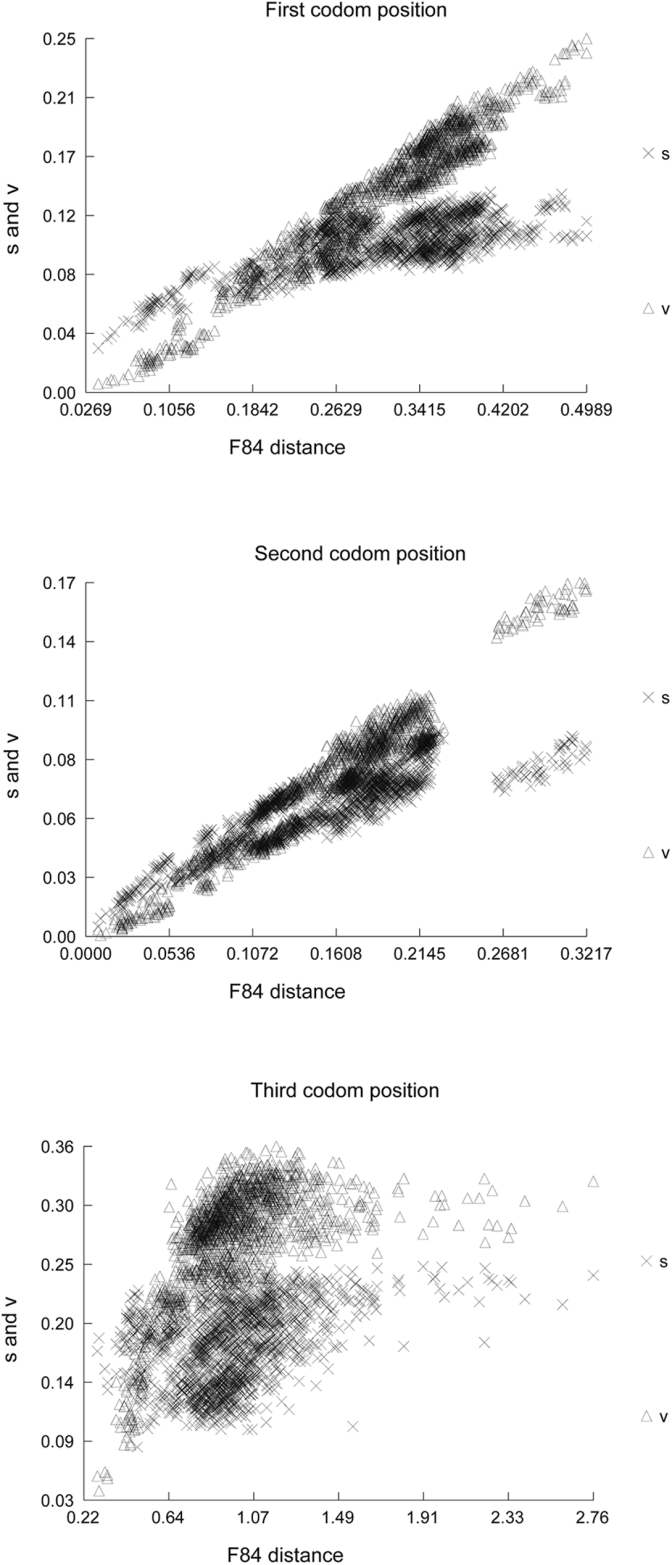
Substitution saturation analysis in the first, second, and third codon positions of 13 PCGs in all analyzed species. S indicates transitions and V indicates transversions.

The saturation analyses on the first, second and third codon positions of the 13 PCGs were showed in Fig 7. Saturation plots indicated substantial substitution saturation in the third codon positions of all PCGs. So, exclusion of the third codon position from protein-coding genes had a considerable improvement in phylogenetic reconstruction. As shown in all Figs, the analyses based on the PCG-123 and ALL-123 performed poorly compared to the PCG-12 and ALL-12, resulting in unique topologies from BI and ML methods. For ALL-123 dataset, the monophyly of Orthoptera is never recovered in ML or BI analyses when compared to ALL-12 (Figs 5 and 6; S3 and S4 Figs).

## Discussion

The newly determined mitogenomes in present study were similar in gene arrangement (Fig 1), nucleotide composition (S1 Table), and pattern of codon usage (S2 Table) when compared to the other available blattarian mitogenomes as well as to the presumed ancestral hexapod [30]. It suggested the conservation of mitochondrial genome evolution within the Blattaria. In contrast, some other dictyopteran insect lineages (termites and mantids) showed a number of variations in gene order or nucleotide composition [48–50]. Lineage-specific purifying selective forces, life history characteristics, or demographic histories may help us to understand the relatively slow rate of evolution in nuclear genes and conserved mitogenome evolution in cockroaches [51]. However, this analysis is preliminary due to the lack of mitochondrial genomes in other major cockroach lineages.

**Fig 8:**
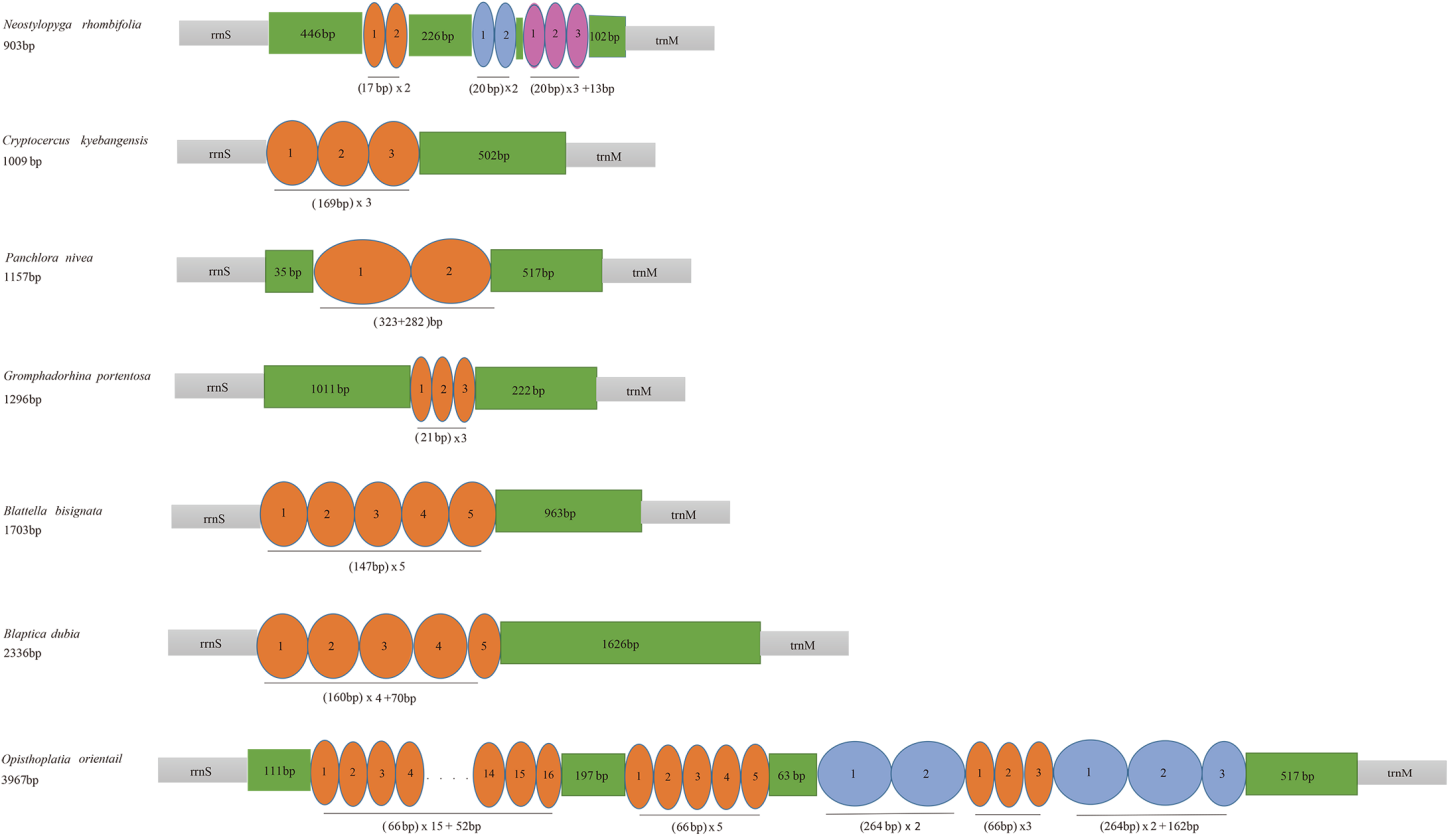
Tandem repeat region of A+T-rich region of six cockroach mitochondrial genomes. The oval with different color indicates tandem repeat sequence. The colored box shows the non-repeat region.

The A+T-rich region known as the control region for insect mitogenome is the largest non-coding region in all blattarian mitogenomes. Because of the various motifs and copies of tandem repeats, the control region exhibits a higher level of sequence and length modifiability than other regions [46]. Among these 14 sequenced blattarian mitogenomes, the lengths of the control regions showed distinctive differences, which ranged from 208bp in *Periplaneta fuliginosa* [24] to 3967bp in *Opisthoplatia or*ientalis [52] (S1 Table). These large length differences mainly result from the absence or presence of tandem repeats and diverse motifs in their control regions. The A+T-rich regions which had comparatively longer sequences contained more and longer tandem repeats (Fig 8). Repetitive sequences of control regions have been used for phylogeographic or population genetics studies. In Isoptera [9], the presence/absence of different repeats could be a marker to resolve the early branching patterns within the Termitidae. Mancini et al [53] reported that the variable number of tandem repeat units were useful for reasoning the genetic structures of populations among closely related taxa. In present study, seven blattarian mitogenome control regions contain tandem repeats, and these tandem repeats appear in dispersed phylogeny positions. Additionally, the homologous alignment among these repetitive sequences of seven blattarian mitogenome control regions revealed a low similarity. None of these repeat units among these seven blattarian mitogenome control regions were sequence homologous and included any conserved sequence (S3 Table). The high sequence diversity between the tandem repeat regions implies that they may have different origin. Besides, if detailed nucleotide divergence of repeat units in more blattarian insect mitogenomes were obtained, these repeat sequences would be probably used for phylogenetic inference and species identification.

Three conserved sequence blocks were identified in the A+T-rich region of *P*. *australasiae* and *N*. *rhombifolia* (Fig 3). The characteristic of CSB1 within the Blattinae did not correspond to the structures previously described in Orthopera and Diptera by Zhang et al [46] because no polythymidine stretch was present. Since our findings showed that the CSB1 occurred in all Blattinae mitogenomes reported and was highly conserved within the Blattinae (95% similarity) (Fig 3B), CSB1 might be a molecular marker to distinguish the Blattinae from other subfamilies. Considering the limited samples, we cannot immediately confirm this block is of functional importance or of identified value for all blattinine species. Separate from CSB1, both CSB2 and CSB3 were common among other dictyopteran insects (Isoptera [9] and Mantodea [50]) as well as among other insect orders such as Lepidoptera [54] and Plecoptera [55]. CSB3 is capable of forming stable stem-loop structure with T-stretch in the loop portion (Fig 4). Since replication has been shown to initiate within or close to stem-loop structures, they may play an important role in regulatory functions during replication as well as transcription of mtDNA [56]. CSB3 also was successfully used as a marker in phylogeny studies. In Cameron’s research [9], they detected stem-loop structures could be molecular synapomorphies within termites. Nevertheless, the presence or absence of stem-loop structures identified in our study is not consistent across clades in our phylogenetic trees. Two aspects should be considered to explain this difference. One involves high nucleotide substitution rate and length mutation rate, which cause highly polymorphic structures in control regions [18]. Another involves sampling number in studies, only some typical species are included in Cameron’s research [9], and additional genera need to be tested to determine the evolution of this feature.

Previous studies have shown insect mitogenomes were the source of sequence data for phylogenetic analysis [17]. Besides, the effectiveness of different analytical approaches was extensively tested [9, 20, 57]. In the present study, the phylogenetic relationships among cockroach families are sensitive to variations in phylogenetic inference methods and different datasets. However, when all genes excluding the third codon positions of PCGs analyzed simultaneously (ALL-12), there was no apparent effect on topology between the optimality criteria analyses. The ALL-12 dataset always recovers the monophyly of Orthoptera, and supports a phylogeny of (((Blattellidae + Bleberidae) + (Polyphagidae + (Blattidae + (Cryptocercidae + Isoptera)))) with high nodal supports (Figs 5 and 6). In fact, when the smaller subsets of data (PCG) are analyzed under different optimality criteria, the effect is more evident in that different topologies and support values were recovered. It indicates rRNA and tRNA genes provide considerable phylogenetic signal, which stabilized the topology structure of phylogenetic tree. Besides, our results showed that the third codon positions of all PCGs were substantial substitution saturation. Compared topologies generated by other datasets, all genes excluding the third codon positions of PCGs could provide better phylogenetic topologies and these topologies were approximately identical to recent studies of Blattaria based on molecular and morphological characters [8,15]. Former study also found that the inclusion of the third codon positions has a negative effect on phylogenetic reconstructions [58]. Therefore, it’s significant to assess the effect on topology of inclusion vs. exclusion of third codons by repetitive analyses in each study.

The phylogenetic analysis with different datasets and inference methods showed some similar topologies among major lineages within the Dictyoptera, and they results strongly supported the monophyly of Blattidae, Blaberidae, Blattellidae, Polyphagidae, as well as the paraphyly of Cryptocercidae + Isoptera (Figs 5 and 6; S3–S8 Figs). Within Dictyoptera, Mantodea was the basal branch in all trees, which has been demonstrated in two studies based on molecular [6] and morphology [12]. However, Lo et al [59] found that *Nocticola* spp. was a sister group of Mantodea with low support value (<50) when Nocticolidae (Blattaria) was added into the dictyopteran phylogenetic analyses (based on mitochondrial COX2, nuclear 18S, and Histone 3 genes). In addition, our phylogenetic analysis showed a strong support for a sister group relationship between termites and *Cryptocercus* species. Although previous researches placed termites outside the cockroaches [60] or even used termites as out-groups [61], resent studies indicated that Isopetera is deeply nested within Blattaria as the sister group of Cryptocercidae based on morphological [12] and molecular data [7]. Our study strongly supported the proposal that Isoptera should be classified as a family (Termitidae), or small set of termite families, within Blattodea, as it was first put forward definitively by Inward et al [62].

The relationships among families and genera of cockroaches were still ambiguous. The placement of Polyphagidae was variable among different datasets and analytical methods. Previous studies also provided different perspective on the position of Polyphagidae. Cheng et al [13] supported that Polyphagidae as the the basal group of Blattodea based on mitochondrial PCGs in NJ and MP analyses. Pellens et al [63] placed Polyphagidae as sister to Cryptocercidae + Isoptera + Blattidae based on a combined data set of 12S, 16S, 18S, and COX2. When Nocticolidae was considered into phylogenetic analyses, the Polyphagidae + Nocticolidae were placed as a sister group to Cryptocercidae + Isoptera (based on five gene loci: COX1, COX2, 16S rRNA, 18S rRNA, and 28S rRNA) [7] or to remaining Blattodea (based on combined dataset of 12S, COX2, 28S, 18S, and histone 3) [62]. These conflicting results about the position of Polyphagidae might be caused by different molecular markers and approaches used in the phylogenetic analyses. Because only one species from the family Polyphagidae was included in the analyses, we could not form a conclusive status for Polyphagidae. The lack of Polyphagidae mitogenomes may lead to some deviations among the Blattaria, so further studies are needed with more diverse species included.

The clade (Blaberidae + Blattellidae) has been called as Blaberoidea, which was supported by many studies [13, 15, 59–61]. Previous studies on the position of Blaberoidea had a variety of conclusions. Most studies sustained Blaberoidea as sister to remaining Blattodea, such as Djernæs et al (based on 5 gene loci) [7], Legendre et al (based on four mitochondrial and two nuclear markers) [15], and Djernæs et al (used both molecular (12S, 16S, COII, 18S, 28S, H3) and morphological characters) [8], but several consistently yielded this clade as sister to Blattidae using mitochondrial COX2 [64], mitochondrial rRNA genes (12S+16S) [60], and 13 mitochondrial PCG genes [13]. Little suggested that Blaberoidea was sister clade to Polyphagidae based on mitocondrial and nuclear genes [14, 65]. It is difficult to assess which phylogenetic scheme is more realistic, but our analysis is more in consistent with the most studies that Blaberoidea as basal clade of Blattodea [63, 66]. Considering these differing research results, the position of Blaberoidea within Blattaria still remains inconclusive and more complete mitogenomes recruited would have a higher probability to resolve the intractable phylogenetic relationship [67–68].

The family Blattidae in this study included one subfamily Blattinae, three genera. An interesting point to consider was that *Shelfordella lateralis* (Shelfordella Adelung, 1910) was inserted in the clade *Periplaneta* (Burmeister 1838), and sister to *Periplaneta americana* in all trees with high support values. This clustering result in present study was in accordance with several previous studies [11, 13, 15, 61, 69], indicating *Shelfordella lateralis* had close affinity with *Periplaneta americana*. Inter-generic variation exceeds intra-generic variation to such an extent that a “barcoding gap” exists can be a good way to delimit genera [70]. Levels of genetic divergence in the COX1 dataset (five *Periplaneta* species and one *Shelfordella lateralis*) were estimated by calculating K2P genetic distances (Table 2). The average interspecific genetic distance within the genus *Periplaneta* was 0.13 (0.076 to 0.170), and the average inter-generic divergence between *Periplaneta* species and *Shelfordella lateralis* was 0.13 (0.126 to 0.163). In Maekawa’s result [11], the K2P distances of COX2 between *Periplaneta* species and *Shelfordella lateralis* (0.119~0.140) were also within intra-generic distance of *Periplaneta* (0.063~0.140). No barcoding gap was detected in either analysis. In addition, female and male genitalia are excellent genetic and specific characters used in categories [6]. All male *Periplaneta* and *Shelfordella lateralis* have symmetrical paraprocts and hypandrium without any armament, and they possess a pair of elongate and fusiform stylis as well as two similarly shaped phallomeres. Historically, this species was originally described as *Periplaneta lateralis*, and the classification of *Shelfordella* questionable [71]. Phylogenetic relationship, genetic distance, and morphological characters suggest that this species should be positioned in the genus *Periplaneta* rather than *Shelfordella* as presently recognized.

## Supporting information

S1 Fig Putative secondary structures of the 22 tRNA genes identified in the mitochondrial genome of *Periplaneta australasiae*. Bars indicate Watson-Crick base pairings, and plus sign between G and U pairs marks canonical base pairings appearing in tRNAs.
(TIF)

S2 Fig Putative secondary structures of the 22 tRNA genes identified in the mitochondrial genome of *Neostylopyga rhombifoli*a. Bars indicate Watson-Crick base pairings, and plus sign between G and U pairs marks canonical base pairings appearing in tRNAs.
(TIF)

S3 Fig Bayesian phylogenetic tree based on the nucleotide data of all genes (BI-ALL-123). Numbers on branches are Bayesian posterior probabilities (BPP).
(TIF)

S4 Fig Maximum likelihood phylogenetic tree based on the nucleotide data of all genes (ML-ALL-123). Numbers on branches are bootstrap proportions (BSP).
(TIF)

S5 Fig Bayesian phylogenetic tree based on the nucleotide data of protein-coding genes (BI-PCG-123). Numbers on branches are Bayesian posterior probabilities (BPP).
(TIF)

S6 Fig Maximum likelihood phylogenetic tree based on the nucleotide data of proten-coding genes (ML-PCG-123). Numbers on branches are bootstrap proportions (BSP).
(TIF)

S7 Fig Bayesian phylogenetic tree based on nucleotide data of protein-coding genes excluding third codon sites (BI-PCG-12). Numbers on branches are Bayesian posterior probabilities (BPP).
(TIF)

S8 Fig Maximum likelihood phylogenetic tree based on the nucleotide data of protein-coding genes excluding third codon sites (ML-PCG-12). Numbers on branches are bootstrap proportions (BSP).
(TIF)

S1 Table Comparisons characteristics of Dictyoptera and other non-endopterygote insect orders mitogenomes. (DOCX)

S2 Table Codon usage in the PCGs of *P*. *australasiae* and *N*. *rhombifolia* mitogenomes. A total of 3721 codons for *P*. *australasiae* and 3722 codons for *N*. *rhombifolia* were analyzed, including the stop codons. RSCU, relative synonymous codon usage. L, L*, S and S* indicate tRNALeu(CUN), tRNALeu(UUR), tRNASer(AGN), and tRNASer(UCN), respectively.
(DOCX)

S3 Table The nucleotide sequences of repeated units in the control regions of blattarian insects. (DOC)
