# Supplementary figures and images for: Complete mitochondrial genomes of two blattid cockroaches, Periplaneta australasiae and Neostylopyga rhombifolia, and phylogenetic relationships within the Blattaria
Source: PLoS One. 2017 May 9;12(5):e0177162. doi: 10.1371/journal.pone.0177162 (PMC5423650; doi:10.1371/journal.pone.0177162)

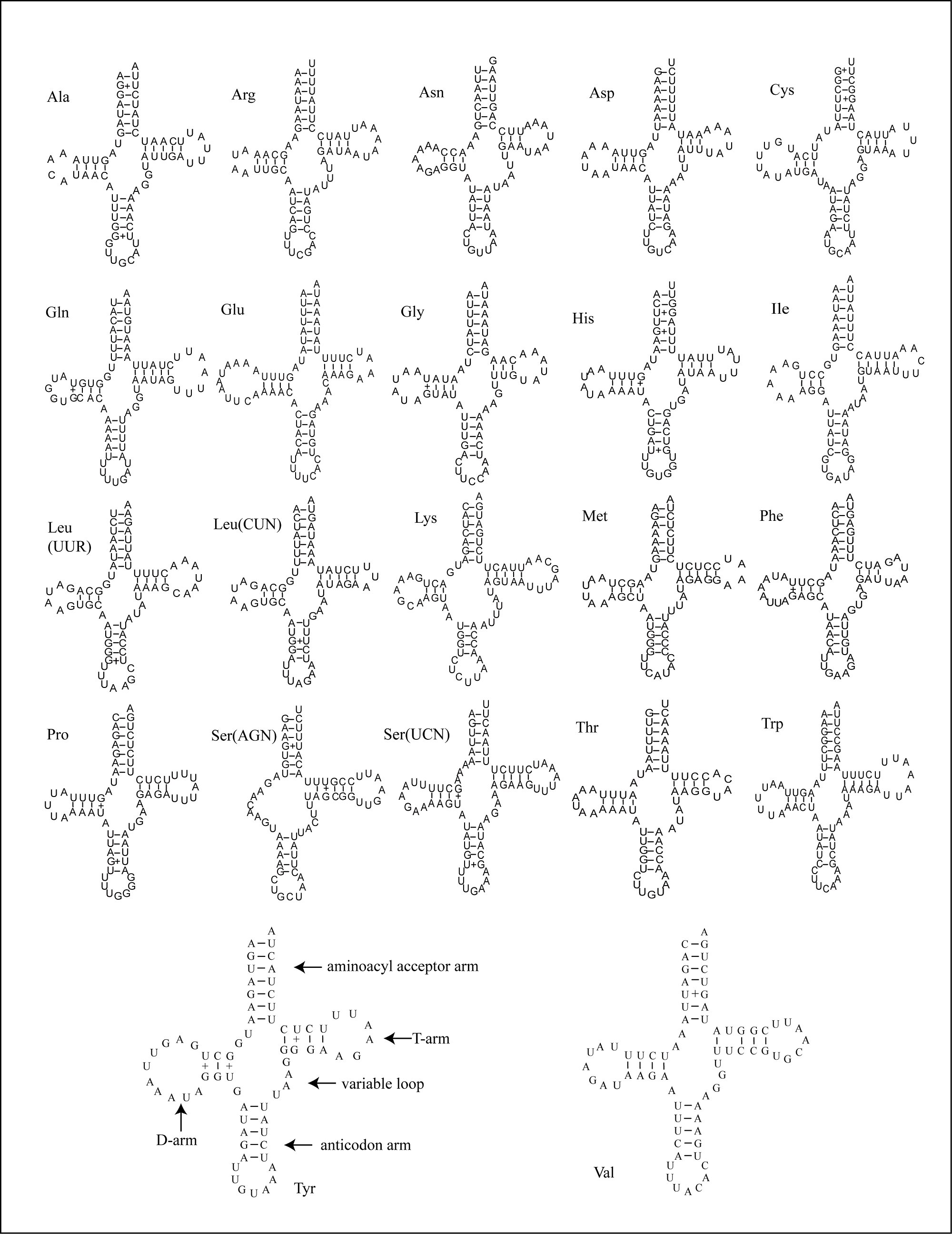

Supplement: S1 Fig — Bars indicate Watson-Crick base pairings, and plus sign between G and U pairs marks canonical base pairings appearing in tRNAs. (TIF) [file pone.0177162.s001.tif]

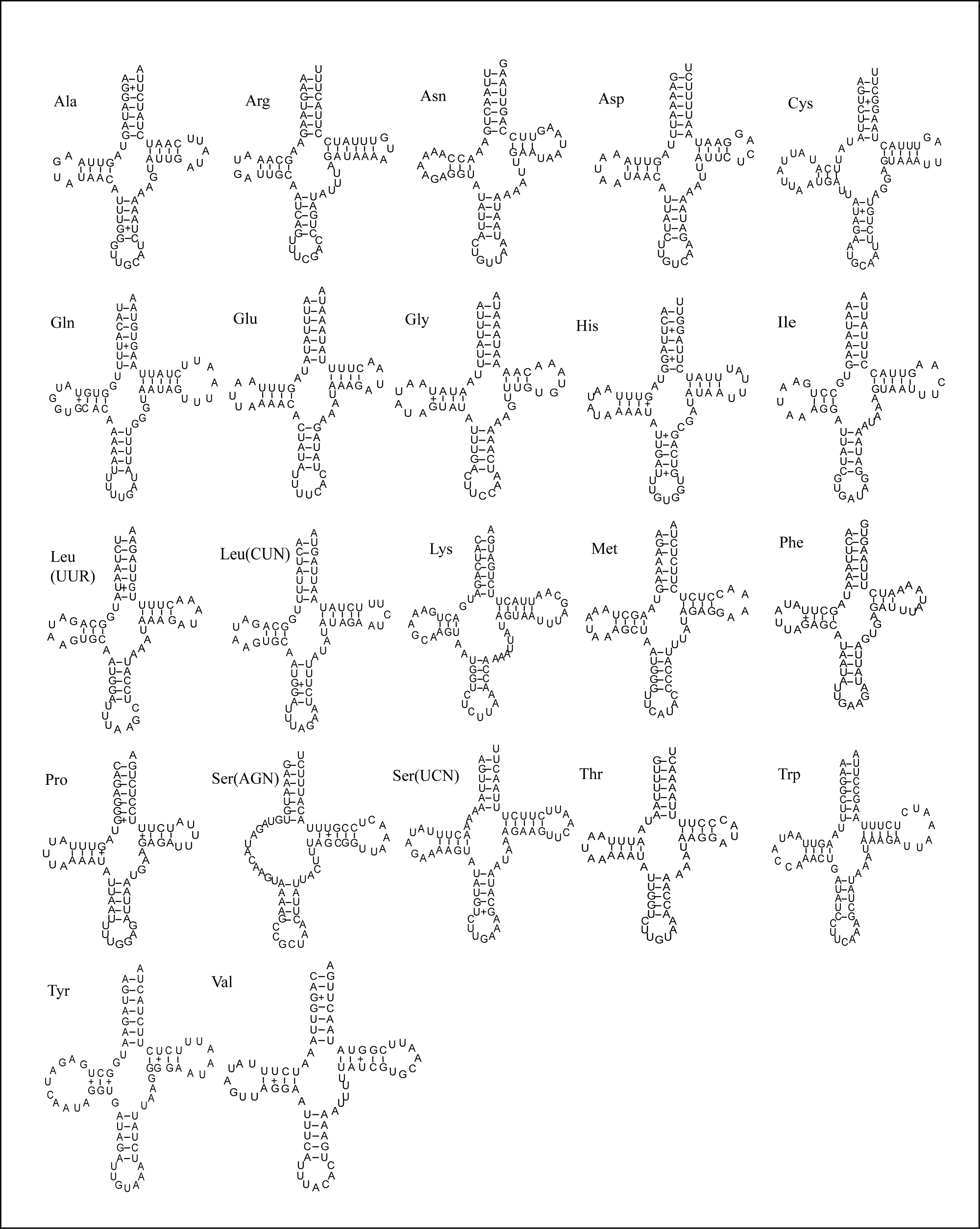

Supplement: S2 Fig — Bars indicate Watson-Crick base pairings, and plus sign between G and U pairs marks canonical base pairings appearing in tRNAs. (TIF) [file pone.0177162.s002.tif]

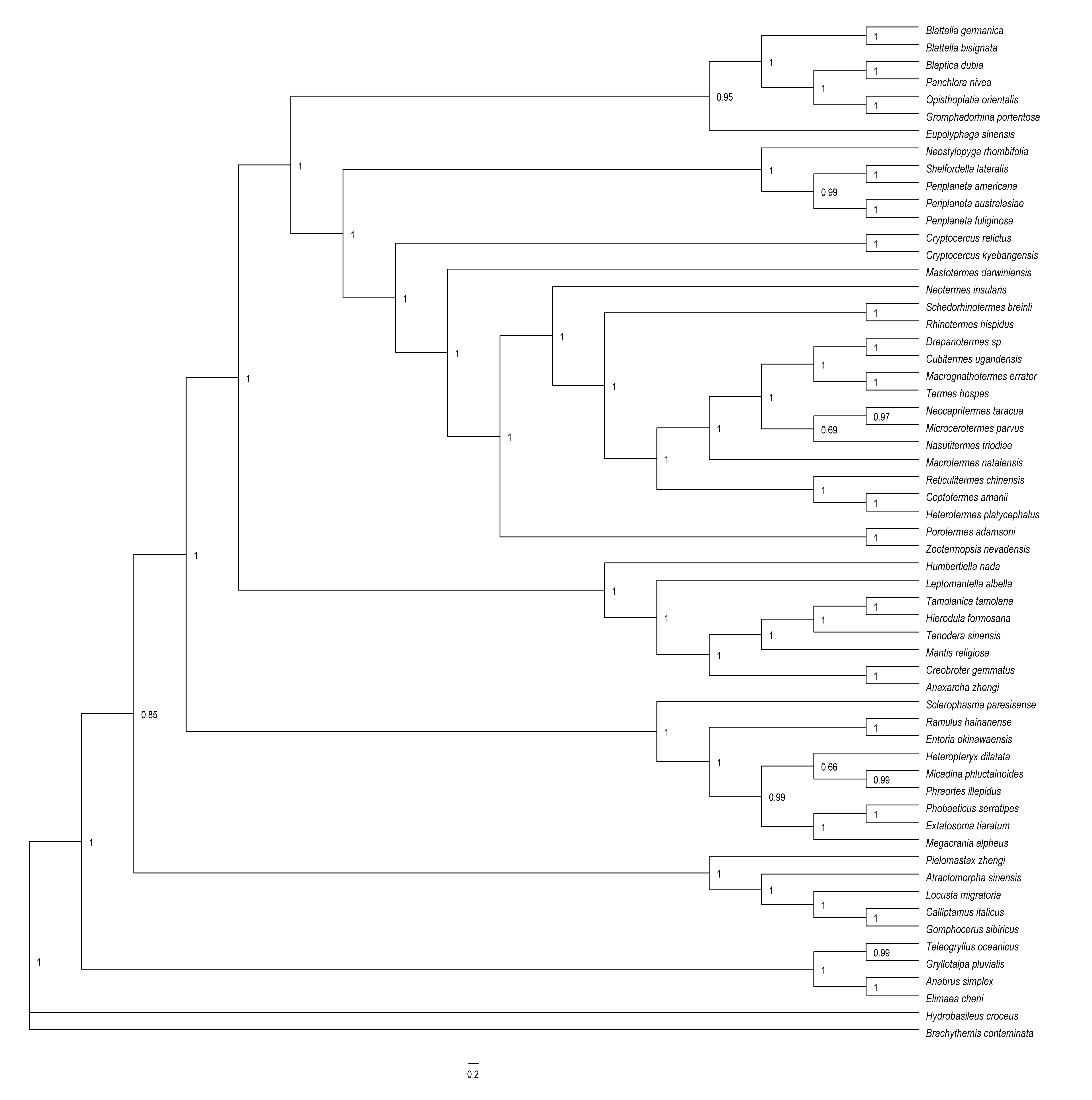

Supplement: S3 Fig — Numbers on branches are Bayesian posterior probabilities (BPP). (TIF) [file pone.0177162.s003.tif]

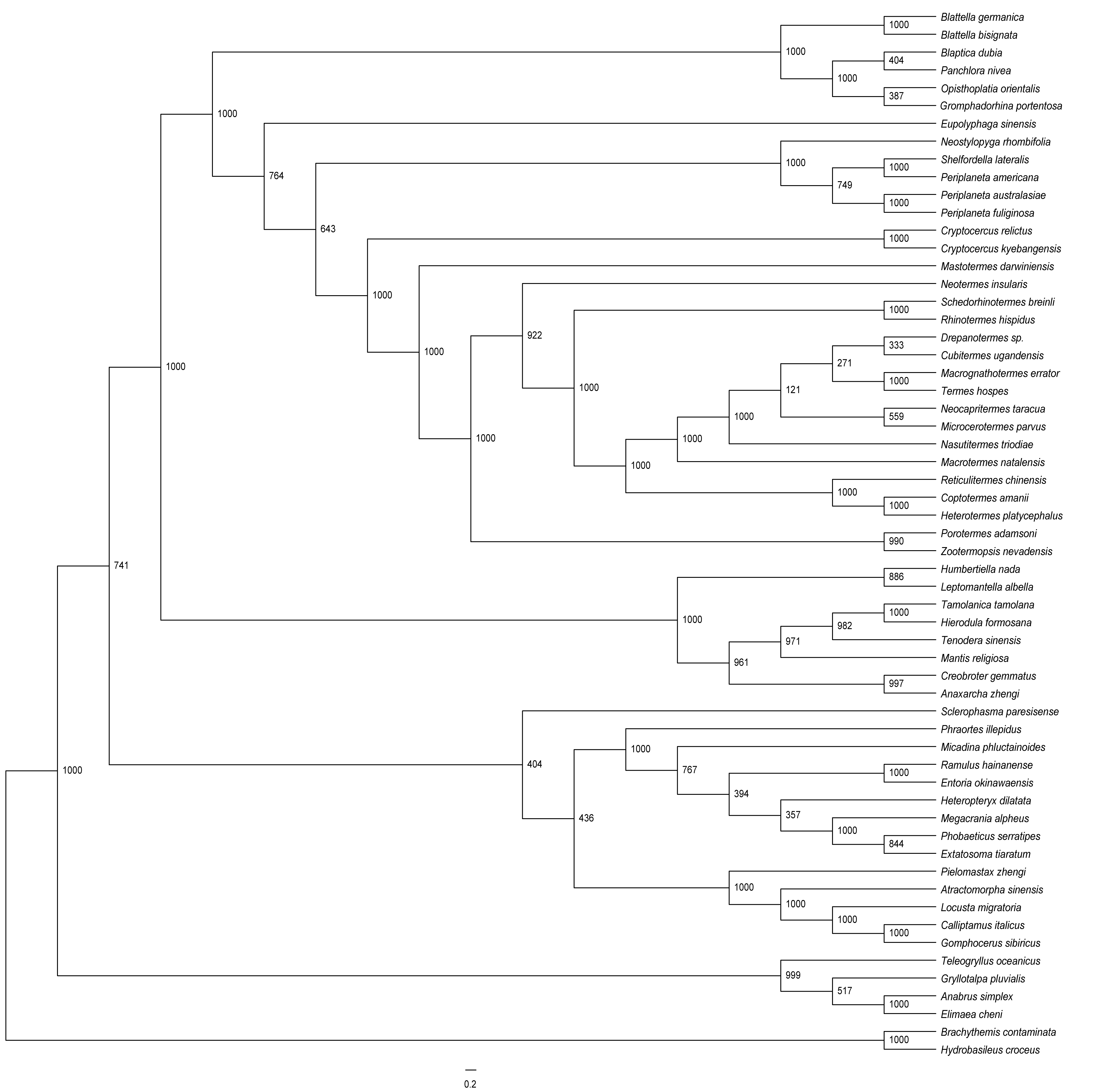

Supplement: S4 Fig — Numbers on branches are bootstrap proportions (BSP). (TIF) [file pone.0177162.s004.tif]

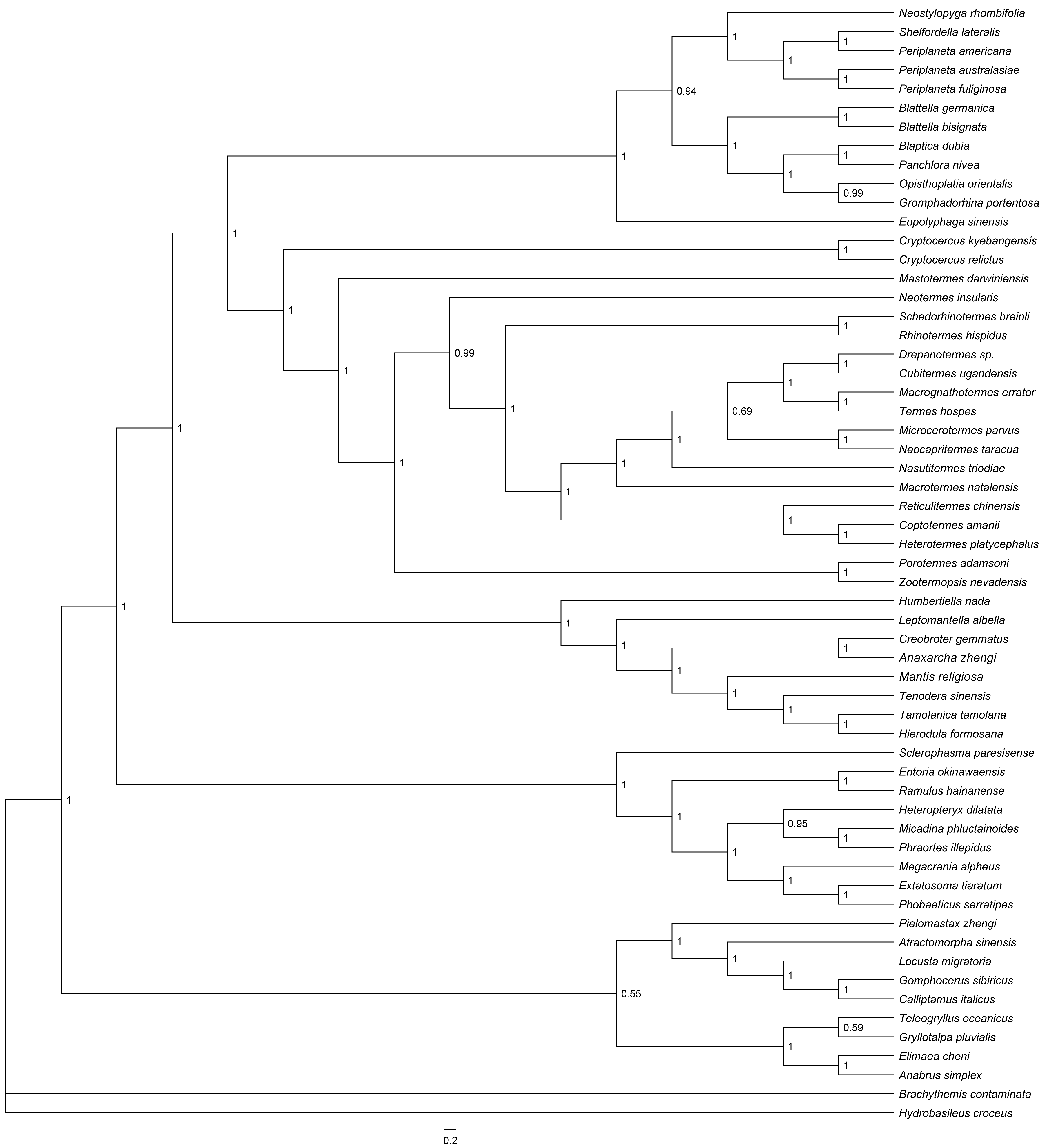

Supplement: S5 Fig — Numbers on branches are Bayesian posterior probabilities (BPP). (TIF) [file pone.0177162.s005.tif]

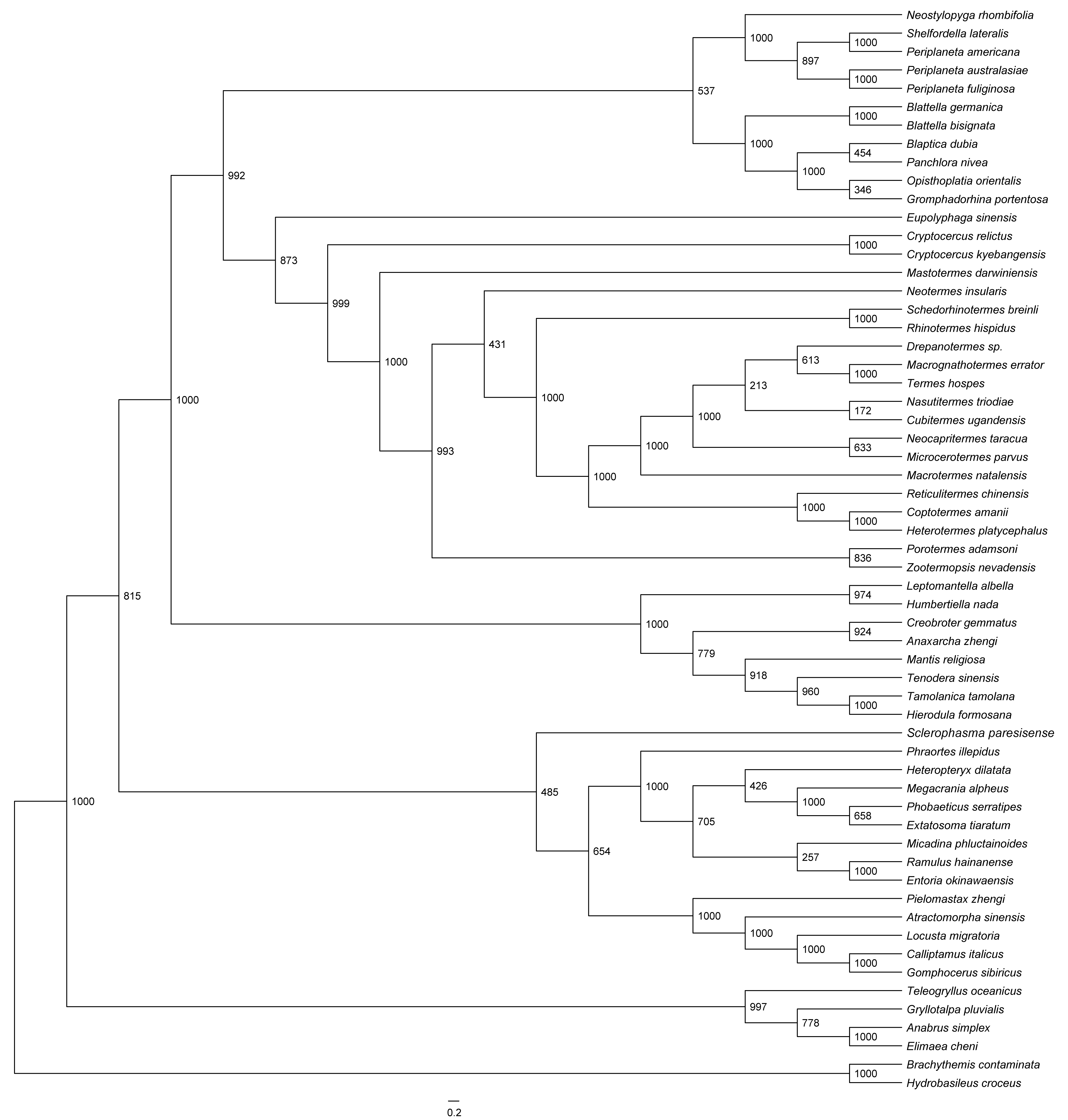

Supplement: S6 Fig — Numbers on branches are bootstrap proportions (BSP). (TIF) [file pone.0177162.s006.tif]

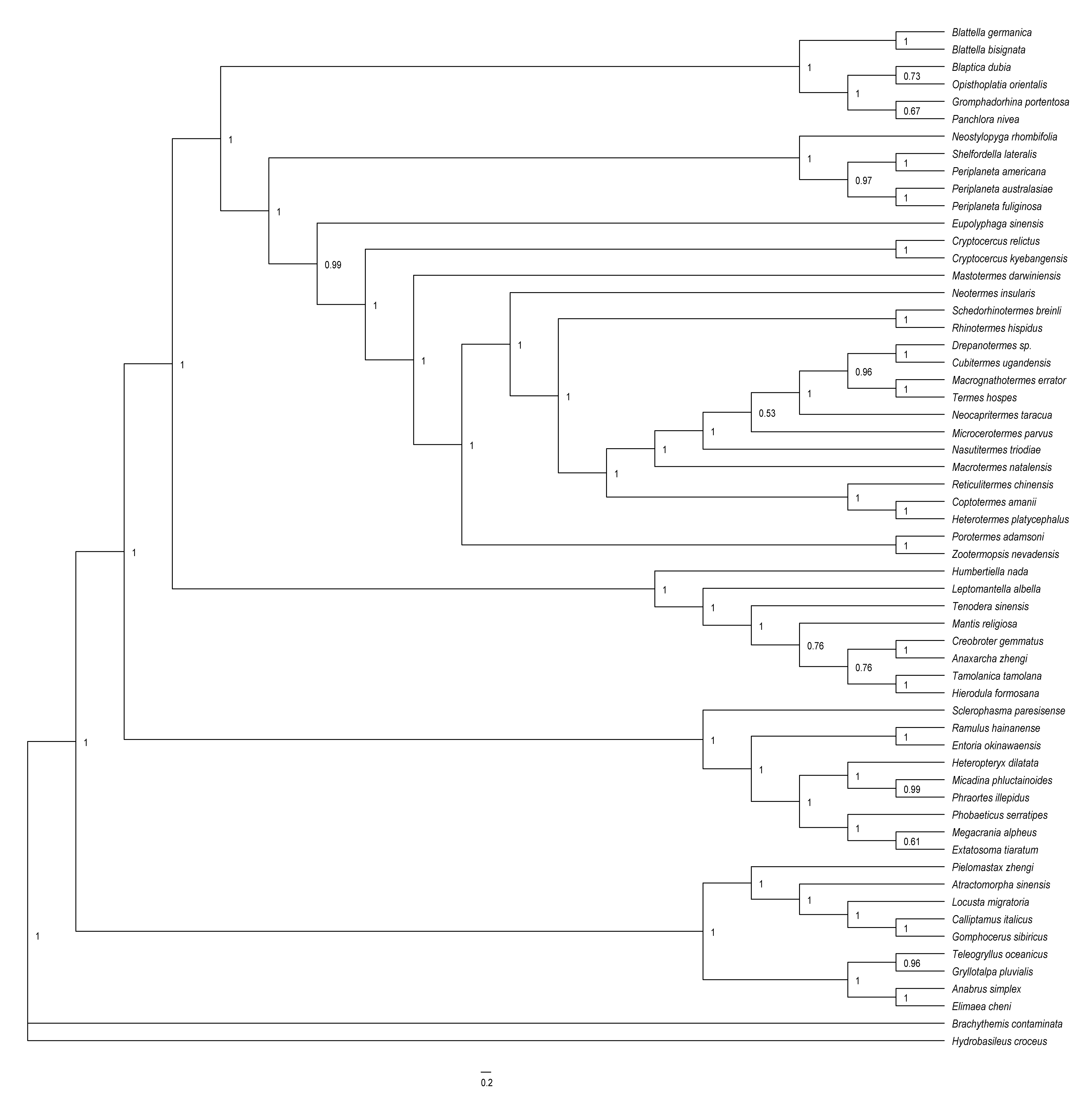

Supplement: S7 Fig — Numbers on branches are Bayesian posterior probabilities (BPP). (TIF) [file pone.0177162.s007.tif]

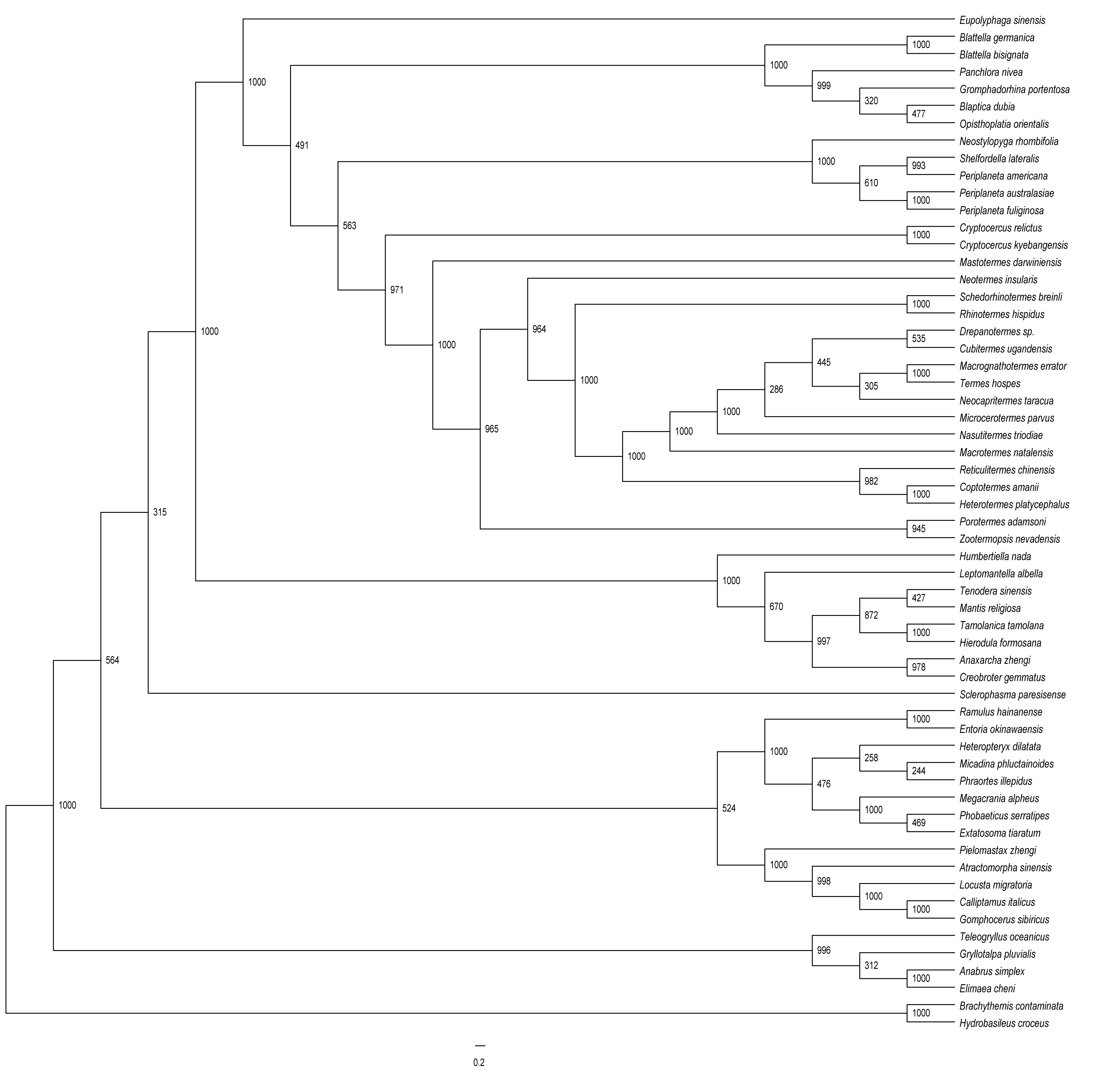

Supplement: S8 Fig — Numbers on branches are bootstrap proportions (BSP). (TIF) [file pone.0177162.s008.tif]
