# Supplementary material for: Complete mitochondrial genomes of two blattid cockroaches, Periplaneta australasiae and Neostylopyga rhombifolia, and phylogenetic relationships within the Blattaria
Source: PLoS One. 2017 May 9;12(5):e0177162. doi: 10.1371/journal.pone.0177162 (PMC5423650; doi:10.1371/journal.pone.0177162)
Supplement: S1 Table — (DOCX) [file pone.0177162.s009.docx]

**S1 Table.** **Comparisons characteristics of Dictyoptera and other non-endopterygote insect orders mitogenomes.**

| **Order** | **Species** | **Whole mitogenome** | | | **PCGs** | | **lrRNA** | | **srRNA** | | **A+T-rich region** | | **Accession No.** |
| --- | --- | --- | --- | --- | --- | --- | --- | --- | --- | --- | --- | --- | --- |
|  |  | Size  (bp) | A+T  (%) | A+T Skew | Codon  no. | A+T  (%) | Size  (bp) | A+T  (%) | Size  (bp) | A+T  (%) | Size  (bp) | A+T  (%) |  |
| Blattaria | *Blaptica dubia* | 17340 | 72.8 | 0.07 | 11145 | 70.8 | 1310 | 75.3 | 814 | 74.0 | 2336 | 78.8 | NC_029224.1 |
|  | *Cryptocercus kyebangensis* | 15720 | 74.4 | 0.24 | 11184 | 73.4 | 1284 | 77.4 | 783 | 75.0 | 1009 | 80.3 | NC_030191.1 |
|  | *Cryptocercus relictus* | 15373 | 73.5 | 0.23 | 11183 | 72.5 | 1281 | 77.0 | 782 | 73.7 | 672 | 80.4 | NC_018132.1 |
|  | *Blattella bisignata* | 16470 | 74.1 | 0.05 | 11141 | 73.7 | 1306 | 77.8 | 790 | 73.2 | 1703 | 74.5 | NC_018549.1 |
|  | *Blattella germanica* | 15025 | 74.6 | 0.05 | 11145 | 74.2 | 1308 | 78.4 | 794 | 73.2 | 253 | 75.1 | NC_012901.1 |
|  | *Eupolyphaga sinensis* | 15553 | 72.0 | 0.12 | 11125 | 70.9 | 1293 | 74.7 | 801 | 72.8 | 857 | 77.8 | NC_014274.1 |
|  | *Gromphadorhina portentosa* | 15992 | 70.8 | 0.03 | 11127 | 69.3 | 1283 | 73.8 | 764 | 70.7 | 1296 | 77.2 | NC_030001.1 |
|  | *Opisthoplatia orientalis* | 18724 | 75.7 | 0.05 | 11142 | 74.4 | 1316 | 78.6 | 780 | 77.2 | 3967 | 77.9 | NC_029225.1 |
|  | *Panchlora nivea* | 16034 | 74.4 | 0.06 | 11166 | 72.9 | 1301 | 78.6 | 776 | 76.0 | 1157 | 82.9 | NC_030002.1 |
|  | *Shelfordella la**teralis* | 15601 | 74.2 | 0.13 | 11167 | 73.1 | 1297 | 78.0 | 800 | 74.0 | 767 | 81.9 | NC_030003.1 |
|  | *Periplaneta fuliginosa* | 14996 | 75.1 | 0.12 | 11167 | 74.6 | 1300 | 78.5 | 801 | 75.8 | 208 | 76.9 | NC_006076.1 |
|  | *Periplaneta americana* | 15584 | 74.2 | 0.13 | 11170 | 73.0 | 1292 | 77.1 | 805 | 74.4 | 764 | 81.5 | NC_016956.1 |
|  | *Periplaneta australasiae* | 15605 | 74.9 | 0.12 | 11164 | 74.1 | 1308 | 77.1 | 812 | 75.1 | 779 | 81.6 | KX640825 |
|  | *Neostylopyga rhombifolia* | 15711 | 74.9 | 0.13 | 11167 | 74.1 | 1296 | 78.7 | 815 | 74.5 | 903 | 80.0 | KX640826 |
| Isoptera | **Coptotermes amanii* | ---- | ---- | ---- | 11175 | 62.6 | 1361 | 67.7 | 813 | 64.8 | ---- | ---- | NC_030011.1 |
|  | **Heterotermes platycephalus* | ---- | ---- | ---- | 11178 | 63.7 | 1365 | 69.6 | 815 | 65.6 | ---- | ---- | NC_030030.1 |
|  | *Reticulitermes chinensis* | 15925 | 65.3 | 0.30 | 11168 | 64.1 | 1301 | 68.9 | 742 | 66.6 | 5656 | 67.0 | NC_025567.1 |
|  | *Rhinotermes hispidus* | 15057 | 66.1 | 0.33 | 11178 | 64.8 | 1388 | 71.0 | 814 | 68.4 | 5501 | 74.5 | NC_030037.1 |
|  | *Porotermes adamsoni* | 16039 | 66.8 | 0.28 | 11161 | 65.7 | 1303 | 70.8 | 801 | 64.8 | 1281 | 71.4 | NC_018121.1 |
|  | *Mastotermes darwiniensis* | 15487 | 68.0 | 0.17 | 11169 | 67.1 | 1301 | 70.9 | 816 | 67.8 | 709 | 72.9 | NC_018120.1 |
|  | *Macrognathotermes errator* | 16330 | 66.8 | 0.27 | 11161 | 65.1 | 1320 | 70.8 | 837 | 67.0 | 1436 | 74.5 | NC_018130.1 |
|  | *Macrotermes natalensis* | 16325 | 65.6 | 0.35 | 11159 | 64.4 | 1310 | 69.0 | 838 | 66.0 | 1419 | 69.3 | NC_025522.1 |
|  | *Microcerotermes parvus* | 16916 | 64.9 | 0.28 | 11169 | 63.1 | 1358 | 69.1 | 814 | 65.6 | 4890 | 69.6 | NC_026114.1 |
|  | *Zootermopsis nevadensis* | 15444 | 69.3 | 0.32 | 11153 | 68.0 | 1291 | 73.0 | 786 | 69.3 | 744 | 77.7 | NC_024658.1 |
|  | *Nasutitermes triodiae* | 15849 | 65.8 | 0.28 | 11167 | 64.4 | 1328 | 70.3 | 819 | 67.3 | 949 | 70.1 | NC_018131.1 |
|  | *Neotermes insularis* | 15799 | 67.8 | 0.26 | 11155 | 67.3 | 1308 | 70.9 | 815 | 67.4 | 985 | 68.5 | NC_018124.1 |
|  | *Neocapritermes taracua* | 16346 | 68.0 | 0.28 | 11169 | 66.5 | 1378 | 71.9 | 811 | 68.8 | 5284 | 74.8 | NC_026116.1 |
|  | *Drepanotermes sp.* | 16542 | 67.2 | 0.26 | 11170 | 65.4 | 1320 | 71.1 | 813 | 66.9 | 1677 | 74.6 | NC_018129.1 |

**S1 Table Continued.**

|  | *Cubitermes ugandensis* | 16491 | 68.4 | 0.25 | 11166 | 66.6 | 1372 | 71.9 | 806 | 68.6 | 4995 | 73.6 | NC_026113.1 |
| --- | --- | --- | --- | --- | --- | --- | --- | --- | --- | --- | --- | --- | --- |
|  | *Termes hospes* | 16461 | 65.9 | 0.28 | 11186 | 64.4 | 1366 | 70.1 | 817 | 66.3 | 4875 | 68.4 | NC_026117.1 |
|  | *Schedorhinotermes breinli* | 15864 | 66.0 | 0.33 | 11177 | 64.0 | 1316 | 72.3 | 823 | 69.0 | 904 | 74.7 | NC_018126.1 |
| Mantodea | *Anaxarcha zhengi* | 16620 | 77.8 | 0.07 | 11161 | 77.0 | 1310 | 80.8 | 795 | 79.4 | 5597 | 79.3 | NC_030268.1 |
|  | *Tenodera sinensis* | 15531 | 75.5 | 0.06 | 11152 | 74.8 | 1311 | 79.1 | 812 | 75.6 | 5631 | 78.9 | NC_030266.1 |
|  | *Creobroter gemmatus* | 15716 | 76.0 | 0.04 | 11150 | 75.5 | 1322 | 79.3 | 783 | 74.1 | 5448 | 79.9 | NC_030267.1 |
|  | *Hierodula formosana* | 16266 | 75.8 | 0.05 | 11151 | 75.3 | 1311 | 79.1 | 803 | 77.0 | 840 | 78.0 | NC_029326.1 |
|  | *Humbertiella nada* | 15866 | 70.1 | 0.01 | 11149 | 68.9 | 1319 | 74.0 | 794 | 71.5 | 5456 | 73.7 | NC_030264.1 |
|  | *Leptomantella albella* | 15534 | 73.7 | 0.02 | 11042 | 72.4 | 1333 | 77.9 | 797 | 74.9 | 684 | 81.9 | NC_024028.1 |
|  | *Mantis religiosa* | 15534 | 76.7 | 0.03 | 11154 | 76.2 | 1317 | 79.3 | 793 | 75.5 | 5561 | 81.1 | NC_030265.1 |
|  | *Tamolanica tamolana* | 16055 | 75.3 | 0.06 | 11151 | 74.8 | 1318 | 77.8 | 777 | 75.7 | 5371 | 74.7 | NC_007702.1 |
| Orthoptera | *Calliptamus italicus* | 15675 | 73.3 | 0.14 | 11185 | 72.5 | 1322 | 75.6 | 801 | 70.5 | 5564 | 86.7 | NC_011305.1 |
|  | *Locusta migratoria* | 15895 | 75.3 | 0.19 | 11216 | 74.2 | 1318 | 78.7 | 831 | 75.5 | 5595 | 84.8 | NC_014891.1 |
|  | *Gomphocerus sibiricus* | 15590 | 74.9 | 0.13 | 11178 | 74.4 | 1314 | 77.2 | 847 | 73.6 | 710 | 83.0 | NC_021103.1 |
|  | *Atractomorpha sinensis* | 15558 | 74.3 | 0.16 | 11163 | 73.6 | 1311 | 76.2 | 819 | 74.8 | 778 | 81.4 | NC_011824.1 |
|  | *Pielomastax zhengi* | 15602 | 71.8 | 0.10 | 11190 | 70.2 | 1301 | 76.4 | 795 | 73.8 | 839 | 80.3 | NC_016182.1 |
|  | *Anabrus simplex* | 15766 | 69.4 | 0.03 | 11198 | 67.7 | 1312 | 72.9 | 785 | 68.9 | 987 | 80.1 | NC_009967.1 |
|  | *Elimaea cheni* | 15831 | 72.6 | 0.02 | 11224 | 71.5 | 1311 | 76.3 | 828 | 74.5 | 999 | 75.5 | NC_014289.1 |
|  | *Teleogryllus oceanicus* | 15660 | 73.0 | 0.11 | 11141 | 72.5 | 1311 | 75.1 | 813 | 71.6 | 5736 | 73.9 | NC_028619.1 |
|  | *Gryllotalpa pluvialis* | 15525 | 72.2 | 0.04 | 11090 | 71.2 | 1236 | 74.9 | 783 | 72.0 | 5798 | 77.7 | NC_011302.1 |
| Mantophasm atodea | *Sclerophasma paresisense* | 15500 | 75.1 | 0.11 | 11160 | 74.1 | 1313 | 77.2 | 781 | 75.3 | 715 | 84.1 | NC_007701.1 |
| Phasmatodea | *Heteropteryx dilatata* | 16618 | 76.1 | 0.19 | 11098 | 75.6 | 1305 | 78.2 | 776 | 75.0 | 1974 | 76.7 | AB477468.1 |
|  | *Extatosoma tiaratum* | 16537 | 76.0 | 0.20 | 11099 | 75.2 | 1299 | 78.3 | 777 | 75.2 | 5823 | 77.2 | NC_017748.1 |
|  | *Megacrania alpheus* | 17124 | 76.9 | 0.20 | 11112 | 75.5 | 1288 | 79.3 | 777 | 76.4 | 2520 | 80.6 | NC_014688.1 |
|  | *Micadina phluctainoides* | 16507 | 78.4 | 0.17 | 11103 | 76.8 | 1284 | 81.2 | 764 | 78.8 | 1842 | 85.0 | NC_014673.1 |
|  | *Entoria okinawaensis* | 16910 | 76.0 | 0.15 | 11101 | 75.7 | 1286 | 79.4 | 780 | 77.7 | 2304 | 73.0 | NC_014694.1 |
|  | *Phobaeticus serratipes* | 16182 | 77.0 | 0.19 | 11068 | 75.9 | 1286 | 80.2 | 758 | 77.6 | 1608 | 80.7 | NC_014678.1 |
|  | *Phraortes illepidus* | 16456 | 77.6 | 0.18 | 11066 | 76.6 | 1279 | 79.9 | 764 | 76.0 | 1903 | 81.7 | NC_014695.1 |
|  | *Ramulus hainanense* | 15590 | 73.1 | 0.18 | 11081 | 71.7 | 1287 | 78.4 | 782 | 76.6 | 374 | 77.0 | NC_013185.1 |
| Odonata | *Brachythemis contaminata* | 15056 | 72.9 | 0.10 | 11087 | 71.8 | 1286 | 77.3 | 799 | 75.8 | 323 | 83.3 | NC_026305.1 |
|  | *Hydrobasileus croceus* | 15088 | 75.0 | 0.09 | 11099 | 74.5 | 1362 | 77.0 | 750 | 74.3 | 5558 | 84.2 | NC_025758.1 |

*, Incomplete mitogenomes lack the entire A+T-rich region. Termination codons were included in 13 PCGs.
