# Supplementary material for: Complete mitochondrial genomes of two blattid cockroaches, Periplaneta australasiae and Neostylopyga rhombifolia, and phylogenetic relationships within the Blattaria
Source: PLoS One. 2017 May 9;12(5):e0177162. doi: 10.1371/journal.pone.0177162 (PMC5423650; doi:10.1371/journal.pone.0177162)
Supplement: S2 Table — A total of 3721 codons for P. australasiae and 3722 codons for N. rhombifolia were analyzed, including the stop codons. RSCU, relative synonymous codon usage. L, L*, S and S* indicate tRNALeu(CUN), tRNALeu(UUR), tRNASer(AGN), and tRNASer(UCN), respectively. (DOCX) [file pone.0177162.s010.docx]

**S2 Table. Codon usage in the PCGs of** ***P. australasiae*** **and *N. rhombifolia* mitogenomes.**

| **Amino** | **Codon** | **RSCU** | | **Amino** | **Codon** | **RSCU** | | **Amino** | **Codon** | **RSCU** | | **Amino** | **Codon** | **RSCU** | |  |
| --- | --- | --- | --- | --- | --- | --- | --- | --- | --- | --- | --- | --- | --- | --- | --- | --- |
|  |  | Pa | Nr |  |  | Pa | Nr |  |  | Pa | Nr |  |  | Pa | Nr | |
| Phe | UUU(F) | 1.54 | 1.56 | Ser* | UCU(S) | 0.91 | 0.87 | Tyr | UAU(Y) | 1.63 | 1.62 | Cys | UGU(C) | 1.33 | 1.20 |  |
|  | UUC(F) | 0.46 | 0.44 |  | UCC(S) | 0.73 | 0.68 |  | UAC(Y) | 0.37 | 0.38 |  | UGC(C) | 0.67 | 0.80 |  |
| Leu* | UUA(L) | 3.69 | 3.44 |  | UCA(S) | 2.14 | 2.03 | Stop | UAA(*) | 1.36 | 1.21 | Trp | UGA(W) | 1.27 | 1.18 |  |
|  | UUG(L) | 0.78 | 0.94 |  | UCG(S) | 0.31 | 0.32 |  | UAG(*) | 0.64 | 0.79 |  | UGG(W) | 0.73 | 0.82 |  |
| Leu(L) | CUU(L) | 0.45 | 0.75 | Pro | CCU(P) | 1.13 | 1.21 | His | CAU(H) | 1.44 | 1.45 | Arg | CGU(R) | 0.51 | 0.74 |  |
|  | CUC(L) | 0.18 | 0.28 |  | CCC(P) | 0.52 | 0.65 |  | CAC(H) | 0.56 | 0.55 |  | CGC(R) | 0.10 | 0.32 |  |
|  | CUA(L) | 0.81 | 0.48 |  | CCA(P) | 2.13 | 2.02 | Gln | CAA(Q) | 1.81 | 1.85 |  | CGA(R) | 2.87 | 2.63 |  |
|  | CUG(L) | 0.08 | 0.11 |  | CCG(P) | 0.22 | 0.12 |  | CAG(Q) | 0.19 | 0.15 |  | CGG(R) | 0.51 | 0.32 |  |
| Ile | AUU(I) | 1.70 | 1.66 | Thr | ACU(T) | 0.95 | 1.03 | Asn | AAU(N) | 1.61 | 1.58 | Ser | AGU(S) | 1.13 | 1.09 |  |
|  | AUC(I) | 0.30 | 0.34 |  | ACC(T) | 0.66 | 0.77 |  | AAC(N) | 0.39 | 0.42 |  | AGC(S) | 0.66 | 0.73 |  |
| Met | AUA(M) | 1.55 | 1.42 |  | ACA(T) | 2.15 | 1.86 | Lys | AAA(K) | 1.51 | 1.46 |  | AGA(S) | 1.04 | 1.09 |  |
|  | AUG(M) | 0.45 | 0.58 |  | ACG(T) | 0.24 | 0.35 |  | AAG(K) | 0.49 | 0.54 |  | AGG(S) | 1.08 | 1.19 |  |
| Val | GUU(V) | 1.69 | 1.35 | Ala | GCU(A) | 1.56 | 1.18 | Asp | GAU(D) | 1.64 | 1.45 | Gly | GGU(G) | 1.10 | 1.10 |  |
|  | GUC(V) | 0.22 | 0.32 |  | GCC(A) | 0.49 | 0.69 |  | GAC(D) | 0.36 | 0.55 |  | GGC(G) | 0.49 | 0.61 |  |
|  | GUA(V) | 1.75 | 2.18 |  | GCA(A) | 1.82 | 2.12 | Glu | GAA(E) | 1.69 | 1.60 |  | GGA(G) | 2.04 | 1.66 |  |
|  | GUG(V) | 0.33 | 0.16 |  | GCG(A) | 0.13 | 0.00 |  | GAG(E) | 0.31 | 0.40 |  | GGG(G) | 0.37 | 0.64 |  |

A total of 3721 codons for *P. australasiae* mitogenome and 3722 codons for *N. rhombifolia* mitogenome were analyzed, including the stop codons. RSCU, relative synonymous codon usage. L, L*, S and S* indicate tRNALeu(CUN), tRNALeu(UUR), tRNASer(AGN), and tRNASer(UCN), respectively.
