# Supplementary material for: Complete mitochondrial genomes of two blattid cockroaches, Periplaneta australasiae and Neostylopyga rhombifolia, and phylogenetic relationships within the Blattaria
Source: PLoS One. 2017 May 9;12(5):e0177162. doi: 10.1371/journal.pone.0177162 (PMC5423650; doi:10.1371/journal.pone.0177162)
Supplement: S3 Table — (DOC) [file pone.0177162.s011.doc]

| **Species** | **Repeated units** | **Sequences** |
| --- | --- | --- |
| *Neostylopyga rhombifolia* | 17bp unit  20bp unit  20bp unit | 5’-ATATAAATTTAATATAA-3’ |
|  |  | 5’-GGTTTTTTTGCGAAAAACCG-3’ |
|  |  | 5’-TTTTTGCGAAAAATTAGGGA-3’ |
| *Cryptocercus kyebangensis* | 169bp unit | 5’-TAAAGTAAAACAAAAATTTCAGGATCACTACCCCAAATAAAGAAACAAATAAAAAAAAATAAAAAAAAGCAACTAATTAAAAATCAAACTTTCCAGCACAAATAAAATTTTTCTTGCCCAACTAAATAGATAAAACTTTTCAAAAATAAAACTAAAAATAAAACTATTA-3’ |
| *Panchlora nivea* | 323bp unit | 5’-TATAGGATCAATTAAACTTATCTAAATATTATTCATCATAATAAAAGTATAAAATTATCATAGGAACCAGCTTTTTAATAAAACAACTATTAACCCCCATTAATAATTATTATTATTAGTGTAAATTTACATATATTTATATTATAGATTACATTCCAATCGATGTTATCTATAAATAATTTAAACTATTAACCCCCATTAATAATTATTATTATTAGTGTAAATTTACATATATTTATATTATAGATTACATTCCAATCGATGTTATCTATAACTAATTTAAACTATTAACCCCCATTAATATTTTAATTTCAAAACCTCTA-3’ |
| *Gromphadorhina portentosa* | 21bp unit | 5’-AAAAATCGGAAAAAAGGGGGT-3’ |
| *Blattella bisignata* | 147bp unit | 5’-ATCTAACAATCAAAAGAACATTTCAAGTGTTAGCAAAACTGTTTTTTACTAATAAATCAAAAAAATAGAGTATAAATTCCTCCCCAAATCCAATAAGCAACCAACTCTTAACCTTCCTAAATTCTTACTTTTTCAACCTTTTTTCAG-3’ |
| *Blaptica dubia* | 160bp unit | 5’-ATCTGTTACGGACAAATAAACAACTTATTTAAAAATACTAAACAAACAAATTCATAATTATAAGAATAACCCTCCCACTCAACAGTAACAAATTACCCCTTTTGTTACTTTTATTCATCATAACCTTCAATGACCCCCATCATTATTGAACTTCACACCT-3’ |
| *Gromphadorhina portentosa* | 66bp unit  264bp unit | 5’-TTATCTAATTGGCAAACCAATAGCTATCTTTATTACCTATTAAAGATAAATCCCCCTTAACTAACT-3’ |
|  |  | 5’-TCTTTATTACCTATTAAAGATAAATCCCCCTTAACTAACTTTATCTAGTTGGCAAACCAATAGTAACATTAATATTACTAATTAAATAATAATTATATAACTAAAATCTTTCATTTAAAATAGATTTTTATCTAATATTAATTTTAATATAATTACCCCCTTTATATAATTAATTACTTACACAAAAGAGTGTATCTGGATTAATATCTAATTATTCTTATTATTTTCTTTATATTATTATATATCAACTTTATAAATATAAAT-3’ |

**S3 Table. The nucleotide sequences of repeated units in the control regions of blattarian insects.**
